# Distinct Cellular Mechanisms Underlie Smooth Muscle Turnover in Vascular Development and Repair

**DOI:** 10.1161/CIRCRESAHA.117.312111

**Published:** 2017-12-12

**Authors:** Urmas Roostalu, Bashar Aldeiri, Alessandra Albertini, Neil Humphreys, Maj Simonsen-Jackson, Jason K.F. Wong, Giulio Cossu

**Affiliations:** From the Manchester Academic Health Science Centre, Division of Cell Matrix Biology and Regenerative Medicine, Faculty of Biology, Medicine and Health, (U.R., B.A., A.A., J.K.F.W., G.C.) and Transgenic Core Research Facility, Faculty of Biology, Medicine and Health (N.H., M.S.-J.), University of Manchester, United Kingdom; and Plastic Surgery Department, Wythenshawe Hospital, Manchester University NHS Foundation Trust, United Kingdom (J.K.F.W.).

**Keywords:** aorta, CRISPR-Cas systems, developmental biology, mice, transgenic, muscle, smooth, vascular

## Abstract

**Rationale:**

Vascular smooth muscle turnover has important implications for blood vessel repair and for the development of cardiovascular diseases, yet lack of specific transgenic animal models has prevented it’s in vivo analysis.

**Objective:**

The objective of this study was to characterize the dynamics and mechanisms of vascular smooth muscle turnover from the earliest stages of embryonic development to arterial repair in the adult.

**Methods and Results:**

We show that CD146 is transiently expressed in vascular smooth muscle development. By using CRISPR-Cas9 genome editing and in vitro smooth muscle differentiation assay, we demonstrate that CD146 regulates the balance between proliferation and differentiation. We developed a triple-transgenic mouse model to map the fate of NG2^+^CD146^+^ immature smooth muscle cells. A series of pulse-chase experiments revealed that the origin of aortic vascular smooth muscle cells can be traced back to progenitor cells that reside in the wall of the dorsal aorta of the embryo at E10.5. A distinct population of CD146^+^ smooth muscle progenitor cells emerges during embryonic development and is maintained postnatally at arterial branch sites. To characterize the contribution of different cell types to arterial repair, we used 2 injury models. In limited wire-induced injury response, existing smooth muscle cells are the primary contributors to neointima formation. In contrast, microanastomosis leads to early smooth muscle death and subsequent colonization of the vascular wall by proliferative adventitial cells that contribute to the repair.

**Conclusions:**

Extensive proliferation of immature smooth muscle cells in the primitive embryonic dorsal aorta establishes the long-lived lineages of smooth muscle cells that make up the wall of the adult aorta. A discrete population of smooth muscle cells forms in the embryo and is postnatally sustained at arterial branch sites. In response to arterial injuries, existing smooth muscle cells give rise to neointima, but on extensive damage, they are replaced by adventitial cells.

## Abstract 2

Supplemental Digital Content is available in the text.

Vascular smooth muscle cells (VSMCs) develop early in the embryo and are essential in regulating vascular tone and strengthening blood vessel walls. The descending aorta arises by fusion of primordial aortae at the midline and as the development proceeds branches out to supply blood to the growing organs. VSMCs are recruited from the surrounding tissues and as such their origin varies depending on the location of the blood vessel.^1,2^ The mechanisms by which VSMCs are recruited and the dynamics of their proliferation are still poorly understood.

**Editorial, see p 194**

**Meet the First Author, see p 186**

Rapid regeneration of smooth muscle coverage is essential for successful repair of vascular injuries. The extent in which the regeneration relies on the proliferation of existing VSMCs and on the recruitment of diverse progenitor cells has been hotly debated.^1,3–5^ VSMCs can in vitro convert from differentiated state into proliferative phase in a process known as phenotypic switching.^6^ Several studies have demonstrated VSMC proliferation and contribution of dedifferentiated VSMCs to neointima formation after vascular injury.^7–12^ Thus, extensive indirect evidence supports the capacity of already differentiated VSMC to re-enter cell cycle. Nevertheless, direct in vivo analysis of this process has been complicated because of the lack of truly VSMC-specific inducible transgenic models. Furthermore, accumulating evidence suggests a great deal of variability in the capacity of VSMCs to respond to injuries.^13^ The underlying cause of this variability has remained unknown.

Various stem and progenitor cells have been described around arteries. Stem cell antigen 1 marks a large proportion of adventitial cells that are capable of giving rise to VSMCs after transplantation to experimental vein grafts.^14^ Similar cells have been identified in human arteries.^15,16^ Adventitial CD34^+^ progenitor cells in rat had minimal role in neointima formation but did provide VSMCs to the outer medial layer of the artery.^17^ A population of adventitial cells expresses mesenchymal stromal cell markers and can contribute to vascular calcification.^18–20^ Lack of cell type–specific marker genes and transgenic models has prevented the construction of a developmental hierarchy of vascular progenitor cells. The diversity of the vascular tree may create different local niches for progenitor cells, influenced by restricted signaling gradients and physical properties. This to date uncharacterized variability may lie behind the variegated response of the vasculature to injuries.

We found that immature VSMCs are marked by high expression of CD146 that controls their maturation. Using a novel transgenic mouse model, we show that adult aorta VSMCs originate from embryonic dorsal aorta resident progenitor cells with limited contribution from surrounding stem or progenitor cells in the following growth period. We describe here a distinct self-renewing population of CD146^+^ progenitor cells at arterial branch points. In response to minor injuries to the artery, VSMCs give rise to the majority of neointima cells, whereas in severe injuries, early VSMC death occurs and is followed by smooth muscle differentiation of adventitial progenitor cells.

## Methods

All developed research models and methods are available on request from the corresponding author. Detailed methods are described in the Online Data Supplement. For adult mice, 2 mg (0.1 mg/g of body weight) of tamoxifen (in corn oil) was injected intraperitoneally for 5 consecutive days. For neonatal labeling, 0.25 mg was injected subcutaneously for 3 days (P6–P8). For embryonic labeling, 3 mg of tamoxifen was injected together with 1.5 mg progesterone into the intraperitoneal cavity of pregnant female. For long-term lineage-mapping, mice were delivered at P20 by caesarean section and fostered. Embryos were fixed in 4% paraformaldehyde for 5 hours For postnatal stages, paraformaldehyde was perfused intracardially, followed by 3-hour incubation of isolated organs in paraformaldehyde.

Fate mapping was performed under Home Office License PPL 70/7435 and vascular injury under PPL 70/8686 according to the UK Animals (Scientific Procedures) Act (1986). Mice were anesthetized with 4% isoflurane with 2 L/min O_2_. The operation site was shaved and cleaned with 0.2% chlorhexidine. An incision was made along the medial aspect of the leg to expose the superficial femoral artery (SFA). The artery was separated from the femoral nerve and vein and controlled using 2-V Acland clamps (S&T, Switzerland) around the site of vascular division. For the supermicroanastomosis model, the artery was repaired at ×40 magnification using an intravascular stenting technique^21^ and 12/0 nylon microsutures (S&T, Switzerland). Vessel patency was checked for good flow through the anastomosis and after repair. Skin and soft tissues were closed with 8/0 nylon sutures (Ethicon, United Kingdom). Wire-induced injury was performed as described.^22,23^ Three millimeter of wire length was inserted into the artery and moved back and forth 10× and retrieved. The arterial wall was repaired by two 12/0 nylon sutures.

Zeiss Axio Imager M2, Zeiss Axio Zoom, and Zeiss Axio Observer.Z1 microscopes were used with Zeiss Zen 2 software. Leica SP5 and SP8 confocal microscopes were used with Leica Application Suite software. Images were acquired at 2× line average and 4× to 6× frame average using sequential scanning mode. Cells were counted in ImageJ 1.51n. Cell counting on histological samples was blinded (counting on Hoechst channel before immunohistochemistry). Statistical analysis was performed in GraphPad Prism 7. Dunnett test was used to analyze statistical significance of the differences between multiple groups. Unpaired 2-tailed *t* test was used to compare 2 data sets.

## Results

**Figure 1.**
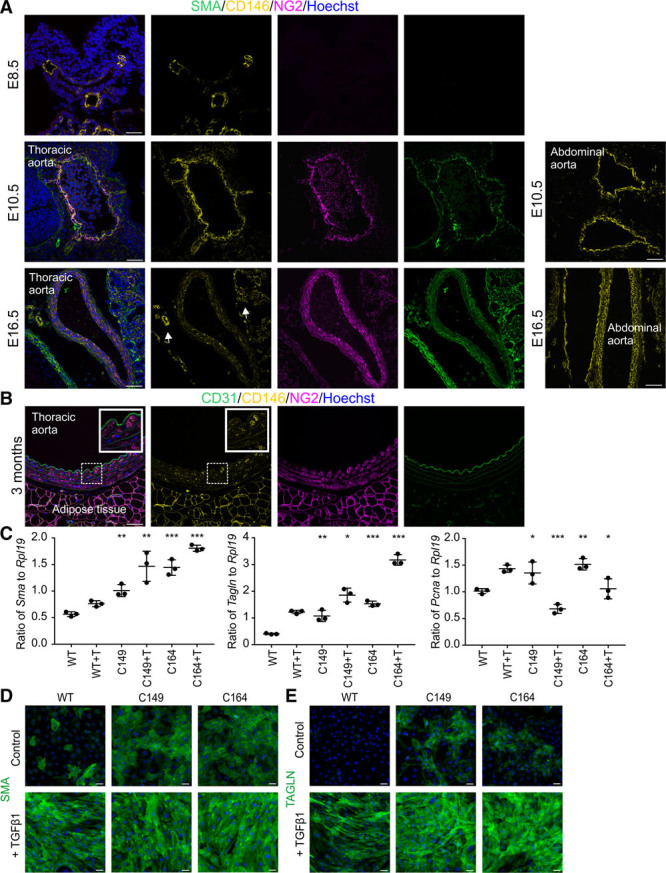
**CD146 is transiently expressed in developing aorta and regulates vascular smooth muscle maturation.** **A**, Descending aorta stained for SMA (smooth muscle α actin), NG2 (neural/glial antigen 2), and CD146 at E8.5, E10.5, and E16.5. CD146 and NG2 are expressed in vascular smooth muscle cells (VSMCs) at E10.5. CD146 expression is downregulated in aortic VSMCs by E16.5 but maintained in smaller arterial branches and microvasculature (**arrows**). Right-hand panels illustrate delayed maturation of the abdominal aorta. At E10.5, it is not completely fused but has CD146^+^ VSMCs. At E16.5, abdominal aorta VSMCs express CD146. **B**, In adult mouse thoracic aorta NG2 is expressed in VSMCs, whereas CD146 shows only scattered expression. CD31 labels endothelium. Boxed area is magnified. Immunohistochemistry was replicated in 4 independent samples. **C**, CRISRP-Cas9 was used to knockout CD146 in 10T1/2 cells, generating cell lines C149 and C164. VSMC differentiation was induced by 48 h transforming growth factor β1 (TGFβ1; +T) exposure. Quantitative reverse transcription polymerase chain reaction was used to characterize expression levels of *Sma* (*Acta2*), *Tagln*, and *Pcna* (proliferating cell nuclear antigen) relative to housekeeping gene *Rpl19* (60S ribosomal protein L19). Biological and technical triplicate, ±SD. Statistical significance was analyzed by Dunnett test by comparing untreated C149 and C164 cells to untreated wild-type (WT) cells and TGFβ1-treated knockout cells to corresponding TGFβ1-treated control cells. Additional data in Online Tables I and II. ***P<0.001, **P<0.01, *P<0.05). CD146 knockout significantly increases smooth muscle contractile protein expression and reduces expression of cell proliferation marker *Pcna*. **D**, Immunocytochemistry illustrates enhanced expression of SMA and (**E**) transgelin (TAGLN) in cell lines C149 and C164. Single confocal plane is shown in **A** and **B**. Scale bars, 50 μm.

Cell adhesion molecules regulate diverse developmental processes. We searched for genes that can uniquely identify developing VSMCs and focused on the expression dynamics of NG2 (neural/glial antigen 2; *Cspg4*) and CD146 (melanoma cell adhesion molecule). NG2 is expressed by VSMCs, glia, myocytes, adipocytes, and chondrocytes.^24–27^ CD146 is expressed by endothelial cells, VSMCs,^28^ and mesenchymal stromal cells.^29^ We found that before the primordial aortae have fused together, at E8.5, they lack NG2^+^ VSMCs while CD146 expression is limited to endothelium (Figure 1A; Online Figure IA). By E10.5, the fusion process is largely completed, and VSMCs can be detected by the expression of smooth muscle α actin, NG2, and CD146. Although NG2 expression is maintained in VSMCs at later time points, the expression of CD146 shows a marked decrease in the descending aorta by E16.5. At the same time, it is maintained in microvascular pericytes and smaller branches of the artery. In adult mouse descending aorta, only scattered expression of CD146 is evident in VSMCs (6.1±2.8% [SD]; n=5; Figure 1B). The development of the abdominal aorta lags behind thoracic aorta (Figure 1A). At E10.5, it has not completed the fusion process although it has acquired CD146^+^ VSMCs. At E16.5, CD146 expression is still uniformly maintained in the abdominal aorta. These results indicate that the transient expression of CD146 marks undifferentiated VSMC progenitors.

Considering the temporally limited expression of CD146 in VSMC development, we next addressed its functional importance in the differentiation process. We used CRISPR-Cas9 to delete CD146 in 10T1/2 cells,^30^ which serve as an in vitro model for VSMC differentiation on transforming growth factor β1 exposure.^31,32^ Wild-type 10T1/2 cells upregulate transgelin (SM22α), smooth muscle α actin, and SMMHC (smooth muscle myosin heavy chain, *Myh11*) after 48 hours exposure to transforming growth factor β1 (Figure 1C–1E; Online Figure IB; Online Tables I and II). Importantly, both CD146 knockout cell lines (C149, C164) showed high level spontaneous expression of characteristic VSMC markers even in the absence of transforming growth factor β1, comparable to unedited cells exposed to differentiation conditions. On exposure to transforming growth factor β1, the mutant cells showed significantly higher expression levels of smooth muscle α actin and transgelin than unedited cells. Both mutant cell lines proliferated less than wild-type cells (Figure 1C; Online Figure IC; Online Tables I and II). These results demonstrate that CD146 regulates the balance between VSMC differentiation and proliferation.

**Figure 2.**
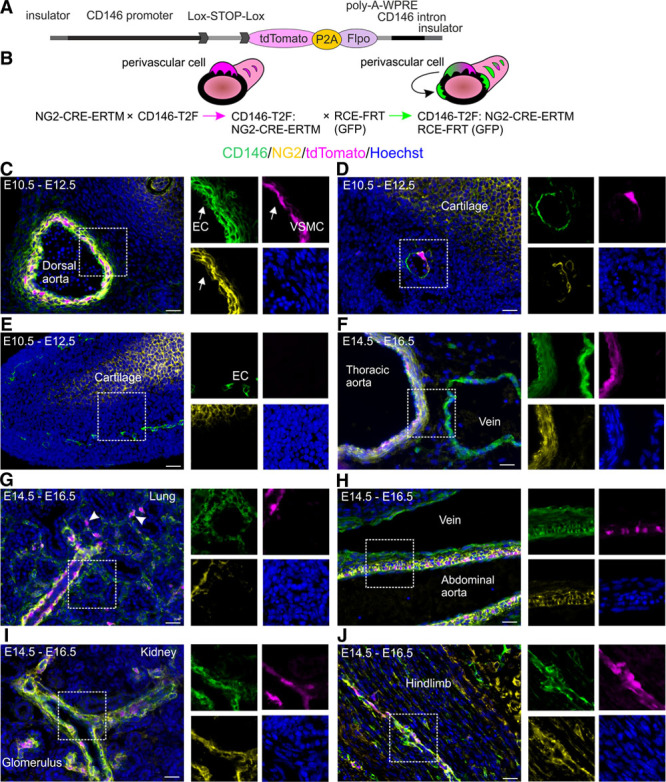
**Generation of mouse model to visualize vascular smooth muscle cell (VSMC) differentiation.** **A**, CD146-T2F transgene DNA construct. **B**, Mating scheme: tamoxifen (TAM) administration in double-transgenic CD146-T2F:NG2-CRE-ERTM line labels CD146^+^NG2^+^ VSMCs with tdTomato. Crossing the offspring to flippase reporter RCE-FRT marks CD146^+^NG2^+^ cells by tdTomato and GFP (green fluorescent protein). Cells that lose the expression of CD146 downregulate tdTomato but remain GFP^+^. **C**–**J**, Analysis of tdTomato expression in the CD146-T2F:NG2-CRE-ERTM line relative to native CD146 (green) and NG2 (neural/glial antigen 2; yellow). Boxed areas magnified on the right. **C**, TAM injection at E10.5 labels CD146^+^NG2^+^ VSMCs in the dorsal aorta by E12.5. Endothelial cells (EC, **arrow**) express only CD146 and remain unlabeled. **D**, tdTomato expression in the femoral artery at E12.5. Limb chondrogenic cells express only NG2 and remain unlabeled. **E**, Microvasculature (endothelium is CD146^+^) in the limb lacks NG2^+^ perivascular cells and tdTomato expression at E12.5. **F**–**J**, TAM injection at E14.5 and analysis at E16.5. **F**, TAM administration at E14.5 labels CD146^+^NG2^+^ VSMCs in the thoracic aorta. NG2 and tdTomato expression are absent from the vein. **G**, tdTomato expression is evident in pulmonary arteries and lung pericytes (arrowhead). **H**, Abdominal aorta is marked by tdTomato, whereas vein remains unlabeled. **I**, tdTomato expression in renal arteries and glomerular perivascular cells. **J**, tdTomato^+^ perivascular cells in limb skeletal muscle at E16.5. Staining pattern was validated in 6 independent samples. All images represent single confocal planes. Scale bars, 25 μm.

Our data indicate that developing VSMCs can accurately be identified by NG2 and CD146 coexpression, whereas other cell types express either one or the other gene separately. We developed a novel VSMC-specific lineage tracing mouse model that relies on sequential activation of 2 recombinases. We fused tdTomato red fluorescent protein^33^ via P2A self-cleaving peptide^34^ to nuclear localized flippase (FLPO)^35^ (Figure 2A) and inserted lox2272^36^-flanked STOP cassette upstream of this sequence. We identified 12 kb region upstream of *CD146/*melanoma cell adhesion molecule in ENCODE data sets^37^ carrying epigenetic signatures of active enhancers (Online Figure IIA). A CCCTC-binding factor enriched region at its 5′ end defines a putative insulator. We synthesized this DNA together with the first *CD146/*melanoma cell adhesion molecule intron, inserted tdTomato-P2A-Flpo cassette in place of the first exon, and placed HS4 insulator on the 3′ side. The linearized DNA was injected to mouse zygotes to generate CD146-T2F transgenic line. When this line is crossed to NG2-CRE-ERTM strain,^38^ the STOP cassette is removed by CRE after tamoxifen administration, initiating the expression of tdTomato and FLPO only in CD146^+^NG2^+^ VSMCs (Figure 2B). By crossing the double-transgenic line to FLPO reporter RCE-FRT,^39^ GFP (green fluorescent protein) expression is triggered by ubiquitous promoter. As a result, CD146^+^NG2^+^ VSMCs are marked by tdTomato and GFP, whereas their CD146^−^ progeny is labeled only by GFP.

We verified the specificity of the double-transgenic line by analyzing the expression of tdTomato relative to native CD146 and NG2. Tamoxifen administration at E10.5 led to tdTomato labeling of VSMCs in the dorsal aorta and femoral artery by E12.5 (Figure 2C and 2D). Because endothelial cells express only CD146 and not NG2, they remained unlabeled (Figure 2C). Likewise, limb chondrogenic cells express only NG2 and not CD146, thus lacking tdTomato expression and proving the specificity of the developed transgenic strategy (Figure 2D and 2E). Early microvasculature lacks NG2^+^ pericytes and tdTomato labeling (Figure 2E). Tamoxifen injection at E14.5 and analysis at E16.5 revealed labeling of CD146^+^NG2^+^ VSMCs in the thoracic aorta but not in the vena cava that lacks NG2^+^ VSMCs (Figure 2F). TdTomato expression was evident in pulmonary arteries, abdominal aorta, renal arteries, and microvascular pericytes (Figure 2G–2J; Online Figure IIB), indicating that the transgene marks VSMCs arising independently of different origins and recapitulates the expression patterns of CD146 and NG2. Embryonic VSMCs express platelet-derived growth factor β that is much more widely spread in the mesoderm (Online Figure IIC). There was no labeling in the lymphatic vessels (Online Figure IID). We calculated recombination efficiency by using double antibody staining (NG2/CD146) in the dorsal aorta at E12.5 and found it to be 62% (±5.2%; SD, n=6), whereas it was 29% (±8.3%; SD, n=6) in the kidney microvasculature at E16.5. An inevitable problem with conditional transgenic models is the leakiness of the STOP cassette. In this case, STOP read-through would label endothelial cells that express CD146 and not NG2. We used fluorescence-activated cell sorter to calculate GFP-marked endothelial cells and found that in most organs <0.2% of endothelial cells expressed GFP, with 3.9% (±1.6%; SD, n=3) being labeled in the skeletal muscle (Online Figure IIE and IIF). These results indicate only limited leakiness.

**Figure 3.**
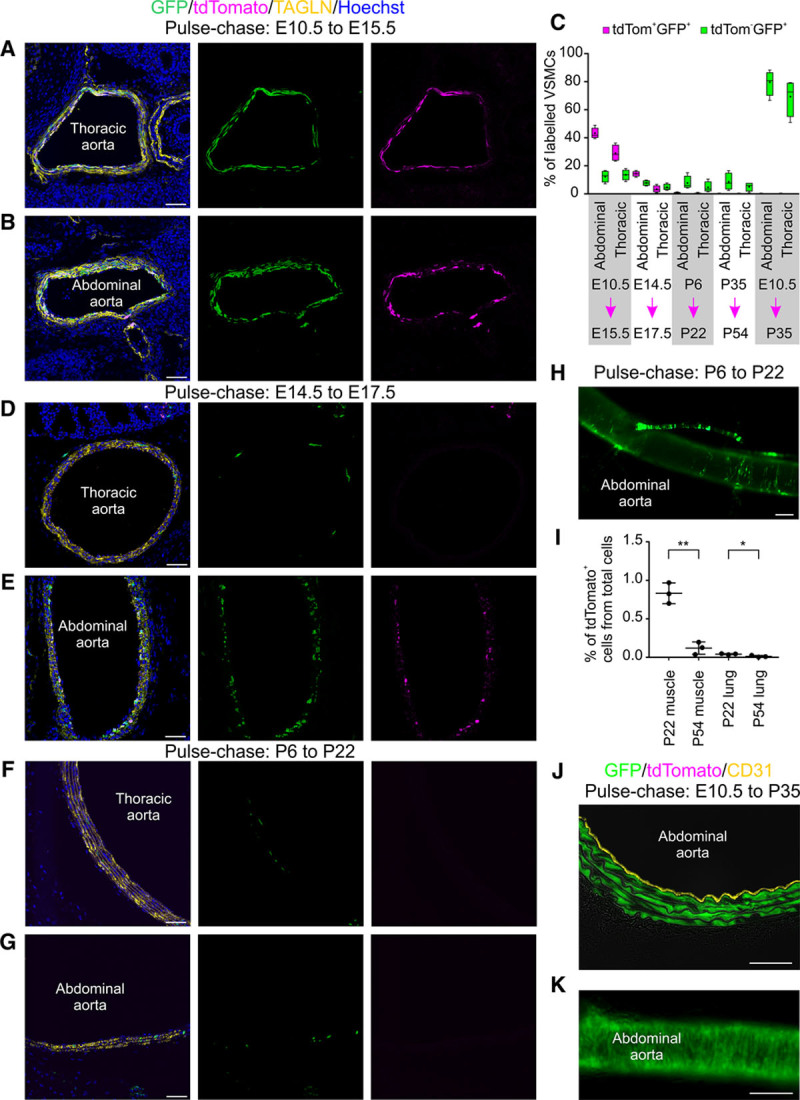
**Early embryonic proliferation of aortic vascular smooth muscle cells (VSMCs) generates the progenitors of adult aorta VSMCs**. Fate mapping analysis was performed with the triple-transgenic strain. VSMCs identified by transgelin (TAGLN) staining. **A**, Tamoxifen (TAM) administration at E10.5 leads by E15.5 to extensive VSMC labeling by tdTomato and GFP (green fluorescent protein) in the thoracic aorta and (**B**) in the abdominal aorta. **C**, Quantification of tdTomato^+^ and GFP^+^ VSMCs at different time points in abdominal and thoracic aortae. Pulse-chase time periods indicated underneath (n=4; box plot with median indicated in boxes with lines, mean values as dots, and whiskers showing highest/lowest values). **D**, TAM pulse at E14.5 leads to scarce labeling of VSMCs in the thoracic aorta at E17.5. **E**, At the same time, strong marking of abdominal aorta VSMCs is visible. **F**–**H**, Postnatal (P6–P8) TAM administration marks few cells in thoracic and abdominal aortae with GFP at P22. **I**, Global organ-level reduction of tdTomato expression can be detected in aging. tdTomato^+^ cells were fluorescence-activated cell sorted from hindlimb skeletal muscles and lung at P22 and P54 (n=3; error bars: SD; unpaired 2-tailed *t* test **P=0.0014, *P=0.022; additional statistics in Online Table III). **J** and **K**, Early embryonic TAM pulse (E10.5) marks VSMC lineages that are maintained to adulthood. Endothelium stained with CD31. Single confocal plane is shown in **A**, **B**, **D**–**G**, and **J**. Scale bars: **A**, **B**, **D**–**G**, and **J**, 50 μm; **H** and **K**, 200 μm.

It is not known when the VSMCs that make up the mature aortic wall populate the vascular niche. We used triple-transgenic strain CD146-T2F:NG2-CRE-ERTM:FRT-GFP to reveal the turnover of CD146^+^NG2^+^ VSMCs. Pulse-chase from E10.5 to E15.5 labeled 29% of all the VSMCs in the thoracic aorta by tdTomato and GFP and 13.5% only by GFP (Figure 3A and 3C). In the abdominal aorta, the respective proportions were 43% and 12.5% (Figure 3B and 3C). This indicates that VSMCs maintain CD146 expression and immature phenotype during embryonic development. Fate mapping analysis spanning late embryonic and fetal stage (E14.5–E17.5) led to reduced labeling, with 3.3% of thoracic aorta VSMC being tdTomato^+^GFP^+^ and 4.8% tdTomato^−^GFP^+^. The transgene expression activity proves cranio-caudal VSMC differentiation because in fetal stage, still 14.3% of VSMC in the abdominal aorta were tdTomato^+^GFP^+^ and 7.8% tdTomato^−^GFP^+^ (Figure 3C–3E; Online Figure IIIA and IIIB). Mice grow rapidly in size, and their body weight increases by ≈5-fold in the first 20 days after birth.^40^ This juvenile growth period was not coupled with reactivation of CD146 expression in the aorta. At P22, <1% of VSMCs in the aortae were marked by tdTomato and <8% by GFP (tamoxifen injection P6–P8, analysis at P22; Figure 3C and 3F–3H; Online Figure IIIC). Transgene expression was still efficiently triggered in smaller arterioles and microvasculature (Online Figure IIID). Limited labeling was evident when tamoxifen was injected in adult mice (5 injections after P35, analysis at P54; Figure 3C; Online Figure IIIE). We detected global organ-level decrease in tdTomato labeling in adult skeletal muscles and lung, suggesting that microvascular pericytes lose CD146 with aging (Figure 3I; Online Figure IIIF). To confirm that the loss of transgene labeling in mature VSMCs is not caused by limited activity of NG2-CRE-ERTM transgene, we crossed NG2-CRE-ERTM line to Rosa26-tdTomato reporter. Tamoxifen administration in these mice (5 injections after P35, analysis at P54) led to prevalent tdTomato expression in the abdominal aorta VSMCs (58.6%±3.4%; SD, n=3; Online Figure IIIG), proving that the decrease in CD146 expression underlies the downregulation of the transgene in mature VSMCs.

CD146^+^ cells make up the majority of embryonic aorta VSMCs, and the transgene does not label neighboring adventitial cells (Figures 2C and 3A and 3B). We asked whether the progeny of the embryonic CD146^+^ VSMCs is maintained in the aorta or replaced during development and postnatal growth. We provided a single tamoxifen pulse at E10.5 and analyzed the aortic wall at P35. We found that the majority of VSMCs were labeled with GFP (79% in the abdominal aorta, 68% in the thoracic aorta; Figure 3C, 3J, and 3K). We conclude that the origin of adult descending aorta VSMCs can be traced back to progenitor cells that exist in the aorta at the time of its development at E10.5.

**Figure 4.**
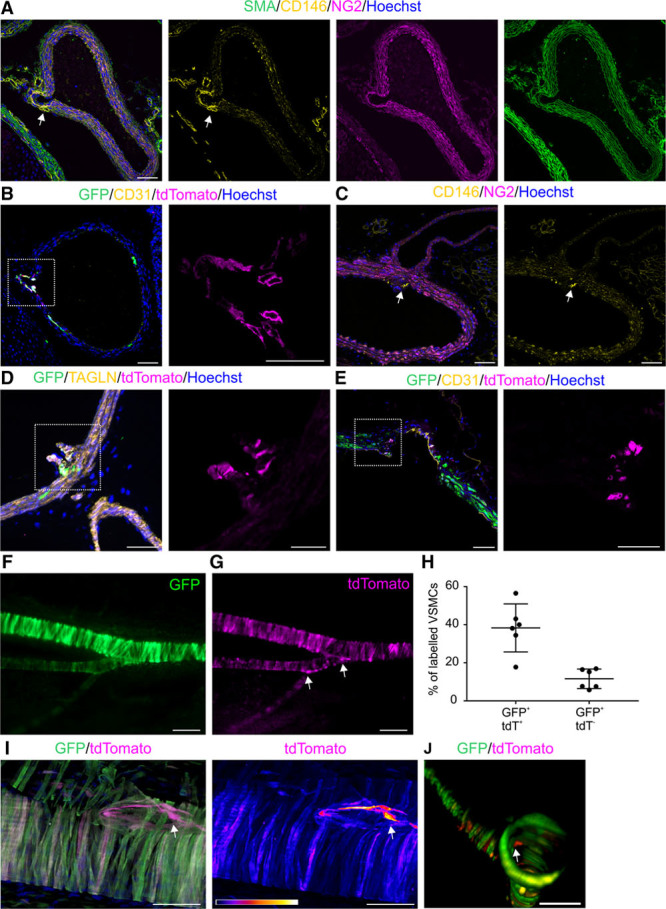
**Immature vascular smooth muscle cells (VSMCs) are maintained at arterial branching sites.** **A**, At E16.5, strong CD146 staining (**arrow**) is evident at intersegmental (intercostal) branching site of thoracic aorta. **B**, Tamoxifen (TAM) pulse at E14.5 labels cells with tdTomato and GFP (green fluorescent protein) at the intersegmental (intercostal) artery branching site of the aorta, whereas few cells are marked in the aortic wall (single channel of the boxed area magnified on the right). **C**, Enhanced CD146 labeling (**arrow**) at renal artery branch site is detectable in 3-mo-old mouse. **D**, Postnatal TAM administration (P6–P8) leads by P22 to tdTomato and GFP labeling at renal artery branching site from the abdominal aorta (boxed area magnified on the right). Branch site cells express transgelin (TAGLN). **E**, Branch site CD146^+^ VSMCs are defined in early embryonic development and maintained to adulthood. TAM was administered at E10.5, and renal artery branch site analyzed at P35 (boxed area magnified on the right). **F** and **G**, TAM administration in adult mice (5 doses at 3 mo, analysis after 2 wk) labels VSMCs in the superficial femoral artery (SFA) with GFP and tdTomato (stereo microscope image). **Arrows**, Elevated tdTomato expression at branch points. **H**, Quantification of GFP^+^tdTomato^+^ and GFP^+^tdTomato^−^ VSMCs in adult mouse SFA (n=6; ±SD). In contrast to the descending aorta, VSMCs are marked by tdTomato in the SFA. **I**, tdTomato is more highly expressed at SFA and descending genicular artery branch site (**arrow**; maximum projection of 3-dimensional [3D] confocal stack). TAM administration as before. Right-hand image demonstrates tdTomato signal intensity in a scale where white indicates strongest signal and dark blue and black lowest signal strength. **J**, TdTomato^+^ cells are visible in the walls of mesenteric artery and are enriched at its jejunal branch site (**arrow**; 3D reconstruction of confocal image stack). Single confocal plane is shown in **A**–**E**. Scale bars: **A**–**E**, **I**, **J**, 50 μm; magnified separate channels of **B**, **D**, **E**, 25 μm; **F** and **G**, 200 μm. NG2 indicates neural/glial antigen 2; and SMA, smooth muscle α actin.

By analyzing embryonic arterial vasculature, we noticed high expression of CD146 in VSMCs at aortic branching sites (Figure 4A). Although tamoxifen administration at E14.5 led to limited labeling of VSMCs in the aortic wall at E17.5, it still labeled VSMCs at aortic branching sites (Figure 4B). Remarkably, these branch site–associated cells maintain high CD146 expression even in the mature aorta (Figure 4C) and consequently are also labeled by tdTomato (Figure 4D; Online Figure IVA). We found tdTomato^+^ branch site–associated cells in adult mice even after a single tamoxifen pulse at E10.5. This indicates that these cells originate early in embryonic development and have long-term self-renewal capacity (Figure 4E). To prove the specificity of the transgene, we generated a second transgenic mouse line from an independent founder and also detected high tdTomato labeling in the branch site–associated progenitor cells (Online Figure IVB). In contrast to the aorta, we found that smaller arteries, like the SFA and mesenteric arteries, are efficiently marked by tdTomato in adulthood (Figure 4F–4H). When tamoxifen was injected in adult mice (5 doses in 3-month-old mice, analysis 2 weeks later), 38.3% of VSMCs in the SFA were tdTomato^+^GFP^+^ and 11.6% tdTomato^−^GFP^+^. Still, even in the SFA and the mesenteric artery, tdTomato was more enriched at arterial branching sites (Figure 4I and 4J). These data show that high CD146 expression identifies a unique cell population at arterial branching sites.

**Figure 5.**
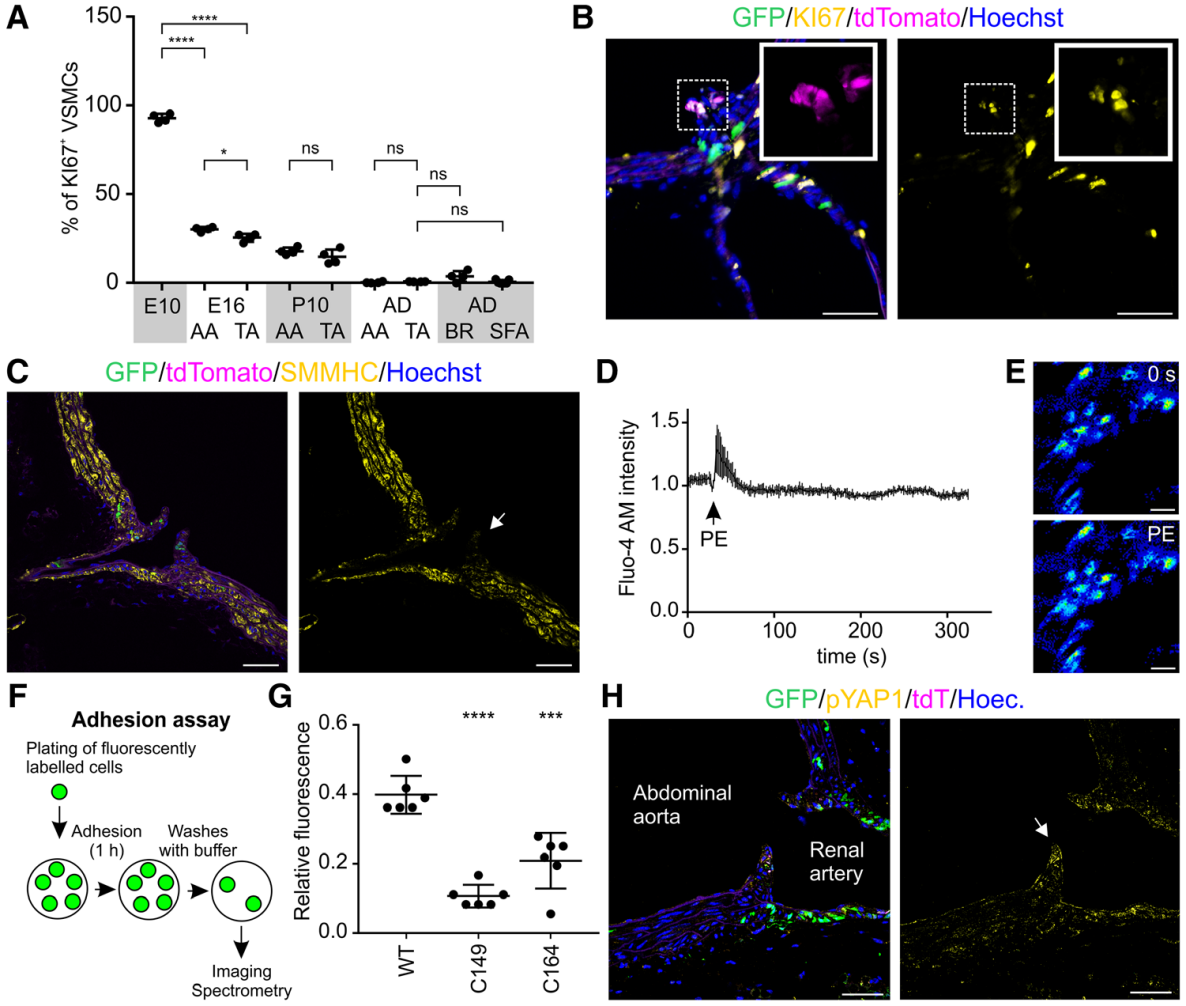
**Functional analysis of CD146^+^ vascular smooth muscle cells (VSMCs).** **A**, The percentage of proliferative VSMCs at E10.5, E16.5, P10, and adult (AD, 3 mo) was quantified by staining for KI67 (n=4, except superficial femoral artery [SFA] n=7; SD is shown). ****P<0.0001, *P<0.05; Dunnett test was used for comparing E10.5 to E16.5 thoracic aorta (TA) and abdominal aorta (AA); unpaired 2-tailed *t* test was used for comparing pairs of samples at later stages; additional statistical data in Online Table IV. **B**, A fraction of TdTomato^+^ progenitor cells at renal artery branch site of the abdominal aorta at P22 are marked by KI67. **C**, Immature VSMCs at intercostal artery branching site show limited expression of SMMHC (smooth muscle myosin heavy chain) in comparison to the aortic wall in adult mouse. **D** and **E**, 10 μmol/L phenylephrine (PE) causes rapid but transient rise in Ca^2+^ concentration in immature VSMCs at mesenteric artery branch site (n=5; SD is shown). Fluo-4 AM dye fluorescence intensity was measured before and after PE addition by using ex vivo confocal imaging. **F**, In vitro cell adhesion assay. Wild-type (WT) 10T1/2 or CD146 knockout cells (C149, C164) were induced to smooth muscle differentiation by 2-d exposure to 5 ng/mL transforming growth factor β1. Cells were trypsinyzed, labeled with green fluorescent cell membrane linker, and allowed to adhere to Matrigel coated surface. After 1 h, the wells were washed 3× with PBS and fluorescence intensity was quantified. **G**, Fluorescence spectrometry quantification of cell adhesion. Background normalized signal intensity with SD is shown (n=6). Dunnett test was used to calculate significance (***P≤0.001; ****P≤0.0001, see also Online Table V). **H**, Aortic branching site (renal artery branching, cushion-like structures indicated by arrow) maintain high expression of phosphorylated YAP1 (yes-associated protein 1) in comparison to mature VSMCs in the aortic wall. Single confocal plane is shown in **B**, **C**, **E**, and **H**. Scale bars: **B**, **C**, **H**, 50 μm; **E**, 25 μm. BR indicates intercostal branch site; and ns, not significant.

We next focused on the functional significance of CD146 expression and the potential role of branch site–associated NG2^+^CD146^+^ cells. We first hypothesized that maintained CD146 expression may correlate with increased cell turnover at arterial branching sites and in smaller arteries. We performed a thorough quantification of KI67 staining at different developmental stages (n=4; average 566 VSMCs analyzed per mouse). These analyses revealed that 93% of VSMCs are proliferative in the aorta at E10.5, whereas by E16.5, there is a significant drop to 30% and 25.5% in the abdominal and thoracic aorta, respectively (Figure 5A; Online Figure VA). This highly proliferative phase coincides with the period when the cell lineages that are maintained to adulthood first emerge. The decrease in cell proliferation occurs earlier in the thoracic aorta, supporting cranio-caudal aorta maturation. At P10, 18% of abdominal aorta VSMCs are proliferative, whereas cell proliferation becomes rare (0.4% in abdominal, 0.8% in thoracic aorta) in 3-month-old mice. We studied VSMC apoptosis by staining for active caspase 3. We analyzed 700 VSMCs in the abdominal and thoracic aortae in 4 mice (5600 VSMCs in total in the descending aortae) but could not detect any apoptotic VSMCs although other cells in the surrounding tissues were labeled (Online Figure VB). These results support limited VSMC turnover in the mature aorta.^41^ We then analyzed aortic branch sites (intercostal artery branching sites from the thoracic aorta) but found no overall significantly higher cell proliferation: 3.7% (SD, 3.03) of branch site–associated VSMCs are KI67^+^ (Figure 5A). There was large variability in cell proliferation across postnatal aortic branching sites, with high proliferative activity in some but no KI67^+^ cells in others (Figure 5A and 5B). This suggests that aortic branch sites do not maintain a constantly elevated cell turnover, yet cell proliferation here may occur in isolated phases. We found that only 0.6% of VSMCs in the SFA were marked by KI67, which is not significantly different from the aorta (Figure 5A; Online Figure VA). Consequently, sustained CD146 expression does not necessarily correlate with higher cell proliferation rate.

CD146^+^NG2^+^ cells cluster in cushion-like structures that extend to the aortic lumen at branching sites. These cells represent immature smooth muscle cells that express low levels of SMMHC (Figure 5C). They share also some morphological similarities with pericytes (small size, cellular processes). VSMCs respond to α_1_-adrenergic receptor agonist phenylephrine by intracellular increase in Ca^2+^ concentration, whereas pericytes often do not show this property.^42,43^ We found that cells at aortic branch site respond to phenylephrine by transient yet modest increase in intracellular Ca^2+^ concentration, indicating that despite expressing low levels of VSMC contractile proteins, they are still capable of responding to vasoconstrictive stimuli (Figure 5D and 5E). Arterial branching sites are exposed to elevated blood flow turbulences, and smaller arteries have to respond to mechanical stretch. We hypothesized that CD146 may, in addition to controlling VSMC maturation, be involved in cellular adhesion to stabilize the vasculature. We confirmed that CD146 loss leads to reduced adhesion of 10T1/2-derived VSMCs (Figure 5F and 5G; Online Figure VC). One of the best characterized mechanotransduction pathways is centered on YAP1 (yes-associated protein 1) signaling,^44^ which intriguingly is one of the few known transcriptional regulators of CD146.^45^ YAP1 mediates VSMC phenotypic switching and is downregulated in VSMC maturation.^46^ This prompted us to study YAP1 expression in the aorta. We found that whereas mature VSMCs express low levels of active phosphorylated YAP1, it is highly expressed at arterial branching sites, providing an insight into how CD146 is maintained at these locations (Figure 5H).

**Figure 6.**
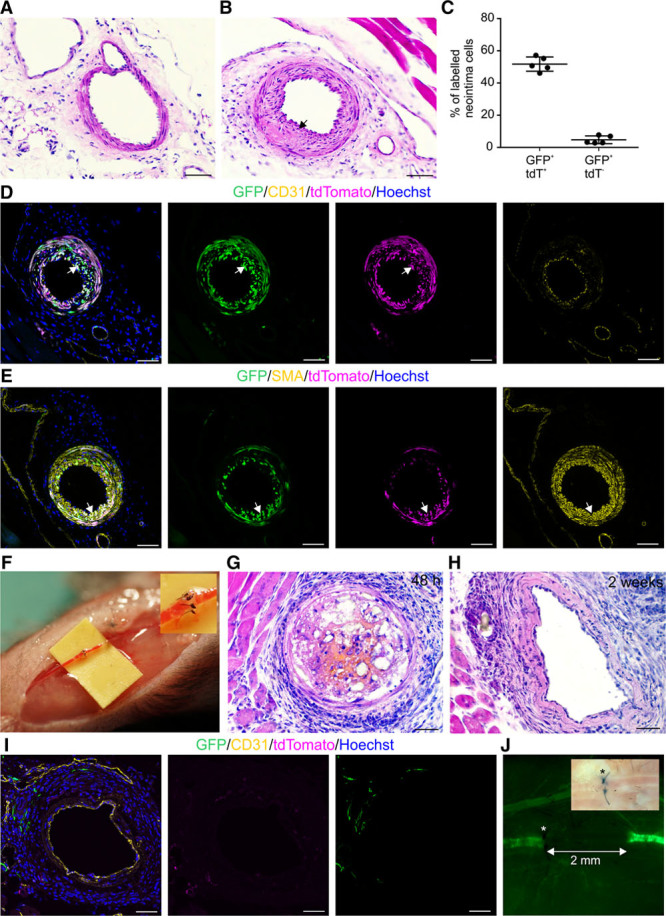
**Distinct response of arterial vascular smooth muscle cells (VSMCs) to minor and major injuries.** **A**, Eosin–hematoxylin staining of healthy superficial femoral artery (SFA). **B**, Neointima is visible (**arrow**) in SFA 3 wk after wire-induced injury. **C**, Quantification of GFP^+^tdTomato^+^ and GFP^+^tdTomato^−^ neointima cells (n=5; ±SD; statistical data in Online Table VI). **D**, GFP^+^tdTomato^+^ neointima cells (**arrow**) surround CD31^+^ endothelium and (**E**) are labeled by SMA (smooth muscle α actin). **F**, SFA supermicroanastomosis by 6 sutures. **G**, Forty-eight hours after anastomosis, transient partial thrombus forms. **H**, Thrombus was not evident at 2 wk after anastomosis. **I** and **J**, Supermicroanastomosis leads to replacement of GFP^+^ VSMCs by 2 wk after surgery. **I**, Intact endothelium is visible 2 wk after anastomosis. **J**, The extent of VSMC replacement indicated with a line. *Site of injury. Bright field image is shown in the upper corner. Single confocal plane is shown in **D**, **E**, and **I**. Scale bars, 50 μm. GFP indicates green fluorescent protein.

The relative contribution of VSMCs and adventitial cells to vascular repair has remained uncertain possibly because of the use of nonspecific transgenic models and variations in injury methods. Because in our triple-transgenic model only VSMCs are labeled in adult mouse SFA, it enables precise fate mapping of VSMCs after vascular injury. We first used established wire-induced injury model that causes damage to the tunica intima.^23^ This model has been widely used to study the formation of neointima, which we confirmed here (Figure 6A and 6B). We induced transgene expression in adult mice (at 2–3 months, 5 daily tamoxifen injections before surgery), inflicted arterial injury, and after 3 weeks analyzed the origin of neointima cells. We found that 51.7% of neointima cells were marked by GFP and tdTomato, whereas 4.8% expressed only GFP (Figure 6C–6E). Considering recombination efficiency, our results indicate that the majority of neointima cells originate from VSMCs and maintain CD146 expression.

We next developed a clinically relevant severe injury and repair model of distal arteries. The SFA with a luminal diameter of 0.1 to 0.3 mm was transected proximal to the popliteal branching site and repaired in a supermicroanatomosis fashion using 6 interrupted 12/0 sutures (Figure 6F; Online Figure VIA and VIB). The complete transection nature of the injury inflicts significant transluminal damage across all layers of the arterial wall similar to what is seen in human arterial injury and repair. All operated mice (n=11) recovered well after the surgery, and we did not observe any difference in limb activity up to the time of tissue retrieval. We analyzed the repair process at early (48 hours; n=5) and late (2 weeks; n=6) time points. We detected partial thrombus formation in all mice at 48 hours after the repair (Figure 6G). Yet, at 2 weeks, no occlusion was evident in 4 of 6 mice (Figure 6H). Remarkably, the SFA wall around the site of anastomosis was devoid of GFP^+^ VSMCs (Figure 6I and 6J). Intact endothelium was present in the operated SFA, and GFP^+^ microvascular pericytes were detected around the site of anastomosis (Figure 6I; Online Figure VIC). Severe vascular injuries are known to cause VSMC apoptosis during the first 2 hours.^47^ We also confirmed loss of VSMC at 48 hours after SFA anastomosis (Online Figure VID and VIE). VSMC death has been proposed to be silent in mice and can leave behind an acellular yet functional vascular wall scaffold.^48^ We, therefore, analyzed whether recellularization and regeneration occur after SFA supermicroanastomis.

**Figure 7.**
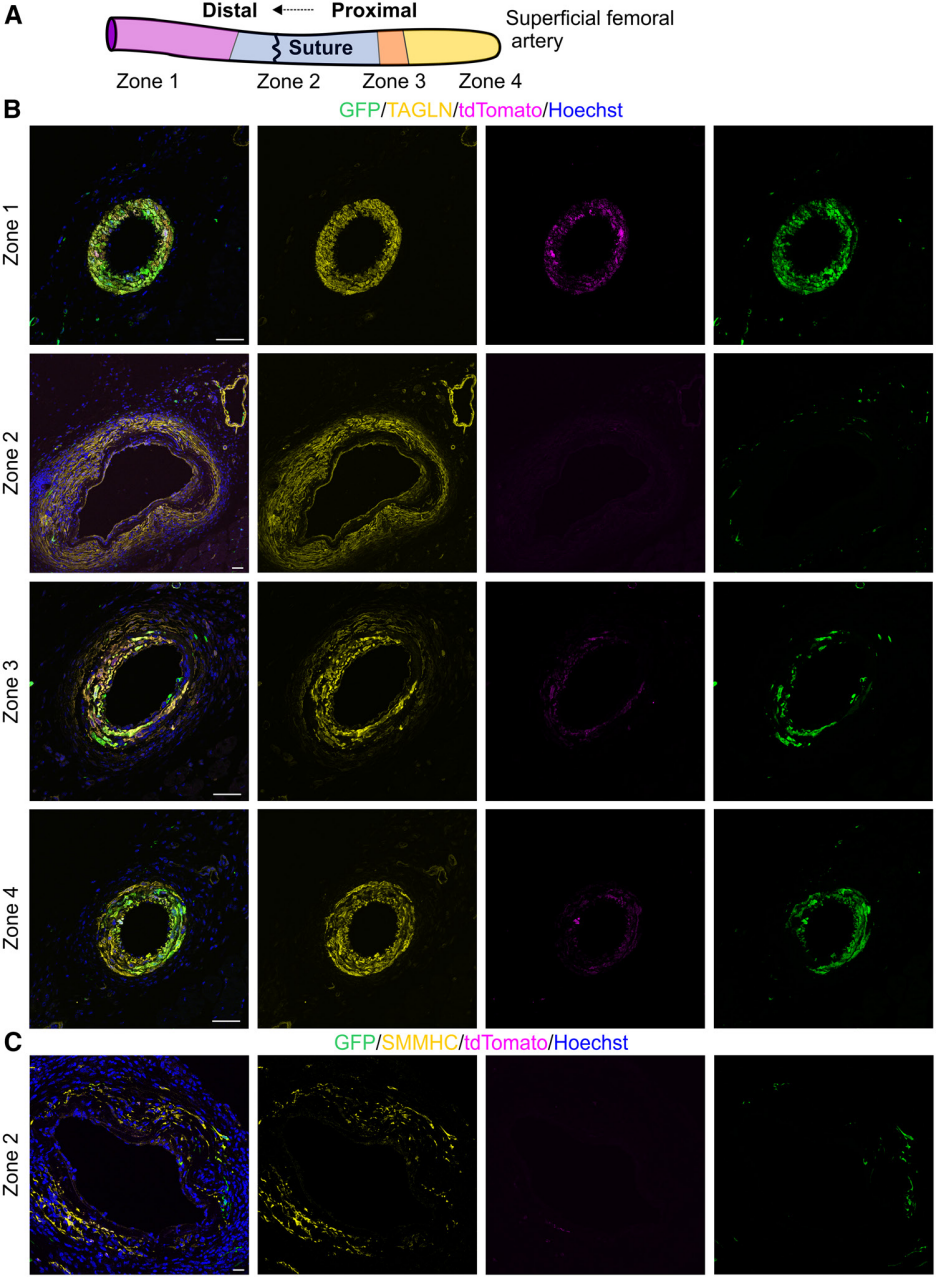
**Spatially determined response to severe arterial injury.** **A**, Schematic of artery with distinct zones relative to the site of microanastomosis. Zone 1 lies distal from the site of anastomosis. Zone 2 encompasses the site of anastomosis. Zones 3 and 4 lie proximal from the site of anastomosis. **B**, Visualization of vascular smooth muscle cell (VSMC) regeneration 2 wk after supermicroanastomosis. Transgelin (TAGLN) identifies VSMCs. Triple-transgenic mice were injected for 5 d with tamoxifen before the surgery. In zone 1, normal vascular wall is evident with GFP^+^tdTomato^+^ VSMCs. In zone 2, expanded TAGLN^+^ area is seen, yet these cells do not express GFP, which is only evident in microvascular pericytes. In zone 3, disorganized layers of tdTomato^+^GFP^+^ cells can be detected. Arterial wall in zone 4 is indistinguishable from healthy artery. **C**, SMMHC (smooth muscle myosin heavy chain) staining is evident in the arterial wall in zone 2. These cells do not arise from GFP^+^ VSMCs. Single confocal plane is shown. Scale bars, 50 μm. GFP indicates green fluorescent protein.

We constructed detailed spatial map of the SFA relative to the site of anastomosis (Figure 7A). In zone 1, distal to the site of injury, the SFA had normal morphology and GFP^+^ transgelin^+^ VSMCs (Figure 7B). Zone 2 encompasses the site of anastomosis and area immediately proximal to it, where GFP^+^ VSMCs were replaced during regeneration. Importantly, 2 weeks after the surgery, the vascular wall had an expanded transgelin^+^ cell layer. These cells showed scattered expression of SMMHC, indicative of terminal VSMC differentiation. Yet, they were not labeled by GFP and thus did not arise from existing VSMCs (Figure 7B and 7C). Zone 3 forms an intermediate area further cranially, where we detected disorganized VSMC layer and GFP^+^ neointima (Figure 7B). In zone 4, the arterial morphology was indistinguishable from healthy artery. These data prove that in severe injury, the pre-existing VSMCs are replaced by a new layer of VSMCs that do not arise from existing VSMCs.

**Figure 8.**
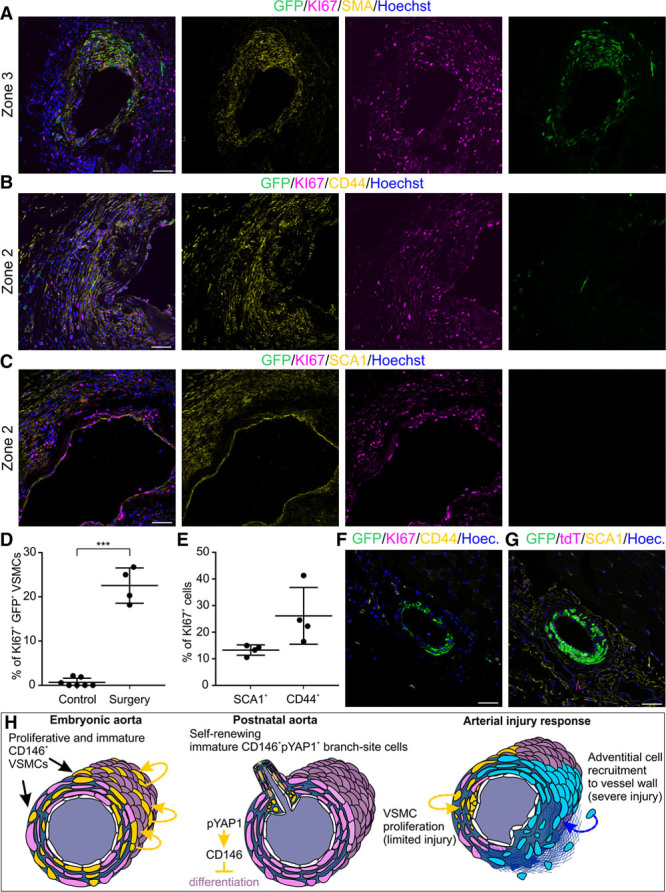
**Adventitial cells proliferate at site of arterial anastomosis and vascular smooth muscle cells (VSMCs) distally from the site of injury.** **A**, After microanastomosis, GFP^+^ (green fluorescent protein) VSMCs proliferate in zone 3. Triple-transgenic 3-mo-old mice were injected with tamoxifen for 5 d before anastomosis and analyzed 2 wk later. KI67 marks proliferative cells and smooth muscle α actin (SMA) VSMCs. **B**, In zone 2, where the injury is most severe, existing VSMCs are replaced by CD44^+^ cells that are frequently KI67^+^. **C**, The adventitial cells are marked by stem cell antigen 1 (SCA1). **D**, Quantification of KI67^+^ GFP^+^ VSMCs in control and operated SFA (***P=0.0044, statistical data in Online Table VII; n=4 for superficial femoral artery [SFA] anastomosis and n=7 for control). **E**, Quantification of proliferative (KI67^+^) CD44^+^ and SCA1^+^ cells at the site of microanastomosis (n=4; ±SD; see also Online Table VIII). **F**, CD44 staining is limited in control SFA. Separate channels are shown in Online Figure VIIA. **G**, SCA1 staining is limited to the adventitia in control artery. Separate channels are shown in Online Figure VIIB. Single confocal plane is shown. **H**, Model for arterial VSMC differentiation dynamics in development and regeneration. In embryonic aorta, CD146 is highly expressed in proliferative VSMC progenitors where it regulates the balance between contractile protein expression and proliferation. Self-renewing CD146^+^ immature VSMCs are maintained in cushion-like structures at postnatal arterial branch sites. Active phosphorylated YAP1 (yes-associated protein 1) in these cells may control CD146 expression and CD146 inhibits terminal differentiation of VSMCs. Unlike the aorta, smaller arteries maintain CD146 expression in adulthood. Arterial wall regeneration after injury involves multiple cell types. Where injury is most severe, the resident VSMCs are replaced by adventitial cells that differentiate into new VSMCs. In case of mild intimal injury, local VSMCs mount a proliferative response and contribute to neointima formation and vascular wall remodeling. Scale bars, 50 μm.

To gain an insight into the origin of the cells participating in arterial repair, we analyzed cell proliferation in the above described zones. We found that in zone 3, 23% of the GFP^+^ VSMCs were proliferative (Figure 8A and 8D). Around the site of anastomosis, where the existing VSMC were replaced, we detected expansion in the cell population characterized by adventitial markers CD44, stem cell antigen 1, and CD34 (Figure 8B, 8C, 8F, and 8G; Online Figure VIIA–VIID). Most notable is the induction of CD44 staining, which is rarely evident in the healthy control artery (Figure 8B and 8F; Online Figure VIIA). We did not detect such intrusion of the intimal layers by adventitial cells after wire-induced injury (Online Figure VIIE and VIIF). The adventitial cells were frequently proliferative 2 weeks after SFA anastomosis: 13.3% of stem cell antigen 1^+^ and 26.2% of CD44^+^ cells stained for KI67 (Figure 8B, 8C, and 8E). These results collectively suggest that new VSMCs may arise from stem cell antigen 1^+^, CD44^+^, and CD34^+^ progenitor cells at the site of anastomosis. We conclude that the degree of VSMCs contribution to vascular repair depends on the severity of injury. Although VSMCs are capable of repairing milder intimal injuries, they fall short when transluminal injury takes place.

## Discussion

VSMCs have major significance in cardiovascular diseases, yet their turnover has remained poorly characterized because of the lack of specific transgenic models. Many cell types are capable of VSMC differentiation and hence uncertainty has persisted on the origin and heterogeneity of VSMCs of the aorta. By developing a novel VSMC-specific lineage tracing model, we demonstrate that adult aorta VSMCs originate from progenitor cells residing in the wall of the aorta already in early embryonic development (Figure 8H). Extensive VSMC proliferation occurs in embryonic period, but only limited cell turnover take place in the adult, which is consistent with VSMCs losing their intrinsic proliferative capacity after birth.^49^ Mature VSMCs in the mouse aorta have an estimated half-life of 270 to 400 days.^50^ These data collectively indicate that VSMCs of the aorta represent long-lived and self-maintaining cell lineages that arise in the early embryo.

We show that CD146 is transiently expressed in VSMCs in the embryonic development of the aorta, whereas smaller arteries maintain its expression to adulthood. We found that CD146 regulates the balance between VSMC proliferation and differentiation. CD146 is a coreceptor for PDGFRβ (platelet-derived growth factor receptor β),^51^ VEGFR2 (vascular endothelial growth factor receptor 2),^52^ binds WNT1 and 5A,^53^ several other ligands, mediates diverse signaling pathways and cell adhesion.^54^ We propose that CD146 fine tunes different signaling cascades to regulate contractile protein levels in VSMCs, thereby enabling more flexible cell shape in developing tissues and arteries. We show that it is also required in VSMC adhesion and may thus strengthen smaller arteries that are affected by movement.

We describe here a hitherto unrecognized population of immature VSMCs that emerges in embryonic development and is confined to aortic branching sites after birth. Since at least 1950s, it has been known that intimal cells form cushion-like structures at arterial branching sites.^55,56^ These sites have in recent years gained considerable attention as they seem to be more susceptible to atherosclerotic lesions.^57^

However, up to now, no single marker gene has been identified for arterial branching sites, and their cellular composition has remained unknown. We show that the unique intimal VSMC population at arterial branching sites is marked by phosphorylated YAP1 and CD146 expression. Because CD146 prevents VSMC differentiation, then these branch site cells express low levels of SMMHC. Yet, they can still respond to contractile stimuli. Considering their undifferentiated state, these cells may contribute to new VSMCs in the growth of lateral branches. However, their primary function in the adult is likely to provide structural support to the arterial branching sites.

Finally, we demonstrate that different cell populations underlie vascular wall remodeling after injury. Various cells have been proposed to contribute to neointima formation, and controversies have persisted largely because of the lack of VSMC-specific fate mapping models. All VSMC contractile proteins and their regulators (SMMHC, smooth muscle α actin, and transgelin) are also expressed in fibroblasts that may arise from different origins and also populate injury sites.^58–60^ We show that in response to limited injury, local VSMCs are the primary contributors to neointima formation, supporting some previous conclusions.^7,8^ Severe injuries lead to early VSMC death, leaving behind extracellular matrix scaffold in arterial wall.^48^ We found that this matrix is repopulated by cells that differentiate into new VSMCs, yet these cells do not originate from existing VSMCs around the injury site, but rather from adventitial cells. Adventitial cells marked by stem cell antigen 1, CD44, CD34, and GLI1 are capable of VSMC differentiation.^14,17,18,61,62^ We propose that differentiated VSMCs cannot respond rapidly to migrate toward injury, and their contribution is therefore limited to local microenvironment.

By using in vivo lineage tracing, we have revealed here the vascular turnover of smooth muscle cells throughout mouse development and postnatal growth, as well as in arterial injury repair. We identified a population of smooth muscle progenitor cells that resides in a specific niche at arterial branch sites.

## Acknowledgments

We thank M.C. Jackson and the Flow Cytometry facility, P. March and the Bioimaging facility, G. Bako, P. Walker, and the Histology core facility (Faculty of Biology, Medicine and Health, Manchester). We are grateful to E. Owen for mouse colony management.

## Sources of Funding

U. Roostalu was supported by Biotechnology and Biological Sciences Research Council Anniversary Future Leader Fellowship (BB/M013170/1). J.K.F. Wong was supported by Medical Research Council (MRC) (MR/M007642/1) and the Royal College of Surgeons of Edinburgh support grants (SRG/14/074 & KAE WONJ4). G. Cossu was supported by British Heart Foundation (PG/14/1/30549), MRC (MR/P016006/1), Duchenne Parent Project (Italy), and Fundació La Marató de TV3 grants.

## Disclosures

None.

## Supplementary Material

## Novelty and Significance

### What Is Known?

- Vascular smooth muscle cells originate from different embryonic cell types.
- Following injury, vascular smooth muscle cells proliferate and contribute to the pathological thickening of the vascular wall.
