# Supplementary material for: Distinct Cellular Mechanisms Underlie Smooth Muscle Turnover in Vascular Development and Repair
Source: Circ Res. 2017 Dec 12;122(2):267–81. doi: 10.1161/CIRCRESAHA.117.312111 (PMC5771686; doi:10.1161/CIRCRESAHA.117.312111)
Supplement: Supplementary file 1 [file res-122-267-s001.pdf]

SUPPLEMENTAL MATERIALS AND METHODS

Cell culture

Authenticated 10T1/2 cells originate from European Collection of Authenticated Cell Cultures, Public Health England (Porton Down, UK) and were cultured in MEM (Gibco, ThermoFisher, Waltham, MA, USA), supplemented with 1% L-Glutamine, 10% foetal bovine serum (FBS), 1% penicillin-streptomycin, 1x MEM Non-Essential Amino Acids Solution. Smooth muscle differentiation was carried out according to published protocol [1]. The cells were serum starved for 24 h before adding medium containing 5 ng/ml recombinant TGFβ1 for 2 days (Cat. No. 7666-MB-005; R&D Systems, Minneapolis, USA). For RNA extraction cells were cultured in 48 well plates and for immunocytochemistry in glass-bottom 24-well Sensoplates (Cat. No. 662892; Greiner Bio-One, Stonehouse, UK).

We have described the generation of CD146 knockout cell lines and their mutations previously [2]. Both C149 and C164 cell lines carry premature STOP codons in the second exon of CD146, encoding the first N-terminal immunoglobulin domain.

Cell adhesion assay

Cells were induced to smooth muscle differentiation as described above, trypsinized and washed once with growth medium containing 10% FBS and twice with medium without serum (each step followed by 5 minute centrifugation at ambient temperature at 500 g). Cells were labelled with PKH67 green fluorescent cell linker (Sigma Aldrich, St. Louis, MO, USA) according to standard protocol after which the cells were washed three times with growth medium to eliminate unbound linkers. Equal amount of cells (30,000) were seeded in 96-well NUNC plate wells (ThermoFisher, Waltham, MA, USA) in 100 µl MEM (Gibco/Thermo Fisher), supplemented with 1% L-Glutamine, 10% FBS, 1% penicillin-streptomycin, 1x MEM Non-Essential Amino Acids Solution. Plates had previously been coated with phenol red free Matrigel matrix (Cornig, New York, USA) according to manufacturer's protocol to achieve thin uniform layer. After 1 h the plates with cells were removed from 37°C incubator, imaged using inverted microscope, and rapidly blotted on paper to remove medium. The wells were washed three times with 37°C PBS and fluorescence intensity was measured with Bio-Rad iMark plate reader (Bio-Rad Laboratories, Hercules, CA, USA). Each cell line was measured in 6 wells, the measurements were normalized to background fluorescence.

Transgenic mouse model generation

The DNA construct for transgene was compiled of several fragments. Ai9 plasmid was a gift from Hongkui Zeng (Allen Institute for Brain Science, Seattle, WA, USA, Addgene plasmid # 22799) [3]. We replaced tdTomato STOP codon in Ai9 by multicloning site, where we inserted P2A-NLS-Flpo (self-cleaving peptide linked to nuclear localized flippase). pFLPo was a gift from Philippe Soriano (Icahn School of Medicine at Mount Sinai, NY, USA, Addgene plasmid # 13792) [4]. We used Ai9 as a template and mutated LoxP sites to Lox2272 sites [5] in order to avoid possible recombination in the triple transgenic mice, as flippase reporter strain RCE-FRT has LoxP site that was used in its generation. We cloned together fragment containing Lox2272-STOP-Lox2272-Kozak-tdTomato-P2A-NLS-Flpo-WPRE-polyA. We next synthesized 12292 bp long DNA fragment corresponding to the upstream genomic region and the first intron of mouse *Mcam* (CD146). It included chicken β-globin HS4 insulator on the 3' side. The synthesized DNA included unique restriction enzyme sites that allowed us to replace the first exon of *Mcam* by the transgene coding construct. The 12 kB long sequence was flanked by restriction enzyme sites that permitted its linearization before pronuclear injection. Thermo Scientific Phusion High-Fidelity

DNA Polymerase (Cat. No. F530L) was used in all the cloning steps, combined with Agilent SURE2 competent cells (Cat. No. 200152; Agilent Technologies Inc., Santa Clara, CA, USA). Qiagen Large-Construct Kit (Cat. No. 12462; Qiagen Gmbh, Hilden, Germany) was used for DNA isolation. Linearized DNA was purified using Qiagen Qiaex II beads (Cat. No. 20021) followed by ethanol precipitation and was eventually resuspended in Millipore EmbryoMax Injection Buffer (Merck Millipore, Billerica, MA, USA). The pronuclear injection (Harlan B6D2F1 (BDF1) wild-type mice) led to 6 founder mice that were back-crossed to C57BL/6 wild-type mice to assess germline penetrance and then to NG2-CRE-ERTM line to study fluorescence specificity and signal strength. Although all founders transmitted the transgene to offspring we detected strongest fluorescence signal in one line that was used for experiments here. The transgene expression did not cause any harmful side effect. All mice were housed in individually ventilated cages (12 h light-dark cycle) and fed standard rodent diet. Sample size for surgical procedures was estimated based on previously found variability. Both male and female mice were used for all experiments and randomly assigned to experimental and control groups. No obvious differences in aortic VSMCs were detected between sexes.

Ex-vivo calcium imaging

3 month old adult mice were sacrificed and the descending aorta was rapidly removed from the abdominal cavity. The aorta was cleaned outside from adipose tissue and cut into smaller segments. Calcium imaging was carried out as previously described, with some modifications [6]. The aorta was stained with Fluo-4 AM dye (Thermo Fisher Cat. No. F14217) in Thermo Fisher Live Cell Imaging Solution (A14291DJ), supplemented with Pluronic F-127 (Thermo Fisher Cat. No. P6866) (7 µl 1 mM Fluo-4 AM, 450 µl imaging solution and 40 µl 10% water-based Pluronic F-127 per aorta). The aorta was incubated with the dye for 1 h at ambient temperature on a horizontal shaker in dark. Following the incubation period the aorta was washed 4 times in imaging solution, cut longitudinally in half and embedded with luminal side up in 1% low melting point agarose (in imaging solution). Phenylephrine hydrochloride (Sigma Aldrich, Cat. No. P6126) was dissolved in imaging solution. Leica SP8 upright confocal microscope with 25x water dip-in objective was used for imaging at 1.48 frame per second speed. Imaging was carried out in Thermo Fisher Live Cell Imaging Solution (containing 1.8 mM $CaCl_2$). Pheylephrine was added to 10 µM final concentration. Fluorescence intensity was quantified in ImageJ for each intimal cell at the aortic branch site (Multi Measure tool for all cells and all time points).

Immunostaining

Samples were fixed in 4% paraformaldehyde (PFA), equilibrated in 15 and 30% Sucrose/PBS, embedded in OCT and cut in cryostat (Leica 3050S) to 7 µm thick sections. Cryosections were washed 3 times in PBS-Tween (0.1%) (PTW), blocked for 1 h in 1% bovine serum albumin (BSA) in PTW and 3 h in 10% donkey serum in PTW. Primary antibodies were incubated overnight at 4°C in 10% donkey serum. On second day the samples were washed 3 times 5 minutes and 3 times 30 minutes in PTW, incubated for 30 minutes in 1% BSA in PTW and for 2 h with secondary antibodies in PTW (ambient temperature, humidified chamber). Following the removal of the secondary antibody the samples were washed 3 times 15 minutes in PTW, 2 times 15 minutes in PBS, rinsed in distilled water and mounted using Vectashield mounting medium (Cat. No. H1000; Vector Laboratories, Burlingame, CA, USA). For staining cells for FACS, the dissociated cells were incubated in PBS and 10% donkey serum on ice for 30 minutes, spun down (700 g, 7 minutes), followed by a 30 minute incubation with primary antibodies diluted in the same buffer. After antibody incubation the cells were washed 3 times in PBS (10% serum). FACS gating was set on non-fluorescent organ samples and unstained fluorescent samples.

For immunocytochemistry cells were washed with PBS and fixed in 4% PFA for 20 minutes. The cells were washed 3 times 15 minutes in PTW and blocked for 1 h in 10% donkey serum in PTW (ambient temperature). Primary antibodies were added overnight in blocking buffer (4°C). On second day (all steps at ambient temperature) the cells were washed 4 times 20 minutes in PTW and blocked for 30 minutes in 1% BSA in PTW. Secondary antibody was added for 1 h after which the cells were washed for 3 times 15 minutes in PTW and kept in PBS for imaging.

FACS

Tissue was diced in Krebs-Ringer-Hepes (KRH), containing 2.5 mM glucose, 2% FBS, 2mg/ml type II Collagenase (Cat. No. LS004176; Worthington Biochemical Corp., Lakewood, NJ, USA) or 1 mg/ml collagenase XI (Sigma Aldrich Cat. No. C7657) for pancreas. Samples were lyzed in shaking water-bath at 37°C until homogenous suspension was reached (40-80 min). Cells were diluted in 5-times volume of ice-cold KRH (2% FBS), passed through 40 μm strainer, centrifuged (700 g, 7 minutes), resuspended in KRH (2% FBS), passed through 40 μm strainer and sorted using BD Bioscience FACSAria.

Antibodies

The following antibodies were used at these indicated concentrations: Rabbit anti-RFP (Rockland 31896; 1:100), Chicken anti-GFP (Abcam Ab13970; 1:100), Rabbit anti-NG2 (Millipore AB5320; 1:100), Rat anti-NG2 (R&D MAB6689-SP; 1:100), Rat anti-CD146 (R&D MAB7718; 1:100), Sheep anti-CD146 (R&D AF6106; 1:500), Rat anti-CD31/PECAM (Dev. Studies Hybridoma Bank; 1:3), Rabbit anti-PDGFRB (Cell signaling 3169; 1:100), Mouse anti-SMA (Sigma A2547; 1:300), Goat anti-SMA (Abcam ab21027; 1:200), Rabbit anti-TAGLN (Abcam ab14106; 1:100), Rabbit anti-SMMHC (Abcam ab53219; 1:100), Rabbit anti-Ki67 (Abcam ab15580; 1:200), Rabbit anti-pYAP1 (Cell Signaling 13008T; 1:100), Rat anti-CD34 (Abcam ab8158; 1:50), Goat anti-SCA1 (R&D AF 1226; 1:30), Rat anti-CD44 (BD 550538; 1:30), Rat anti-CD31-Alexa647 (BD 563608, 1:200). Diverse Alexa-Fluor or DyLight conjugated secondary antibodies from Thermo Fisher Scientific were used at 1:500 dilution.

RNA isolation and RT-qPCR

RNA was extracted using TRIzol reagent (Cat. No. 15596018; Thermo Fisher Scientific) according to manufacturer's protocol and resuspended in 20 μl water. RNA was quantified with Nanodrop 2000 and equalized to same concentration before cDNA synthesis. Reverse-transcription was performed using random hexamer primers and RevertAid First Strand cDNA Synthesis Kit (Cat. No. K1622; Thermo Fisher Scientific). RT-qPCR was carried out using FastStart Essential DNA Master Mix (Cat. No. 06402712001; Roche diagnostics GmbH, Mannheim, Germany) on a Roche LightCycler 96 system. RT-qPCR was performed in technical and biological triplicate for each sample, using standard dilution series on every plate. Roche LightCycler software version 1.5 was used for quantification. Primer sequences were acquired from Harvard PrimerBank (Spandidos et al., 2008; Spandidos et al., 2010; Wang and Seed, 2003): *Acta2* (ID 6671507a1); *Pcna* (ID 7242171a1); *Rpl19* (ID 6677773a1); *Tagln* (ID 6755714a1).

FActa2 GTCCCAGACATCAGGGAGTAA, RActa2 TCGGATACTTCAGCGTCAGGA
FPcna TTTGAGGCACGCCTGATCC, RPcna GGAGACGTGAGACGAGTCCAT
FRpl19 ATGAGTATGCTCAGGCTACAGA, RRpl19 GCATTGGCGATTTCATTGGTC
FTagln CAACAAGGGTCCATCCTACGG, RTagln ATCTGGGCGGCCTACATCA

SUPPLEMENTAL REFERENCES

1. Shi N, Chen SY. Cell division cycle 7 mediates transforming growth factor-beta-induced smooth muscle maturation through activation of myocardin gene transcription. *J Biol Chem*. 2013;288:34336-34342
2. Moreno-Fortuny A, Bragg L, Cossu G, Roostalu U. Mcam contributes to the establishment of cell autonomous polarity in myogenic and chondrogenic differentiation. *Biol Open*. 2017
3. Madisen L, Zwingman TA, Sunkin SM, Oh SW, Zariwala HA, Gu H, Ng LL, Palmiter RD, Hawrylycz MJ, Jones AR, Lein ES, Zeng H. A robust and high-throughput cre reporting and characterization system for the whole mouse brain. *Nat Neurosci*. 2010;13:133-140
4. Raymond CS, Soriano P. High-efficiency flp and phic31 site-specific recombination in mammalian cells. *PLoS One*. 2007;2:e162
5. Lee G, Saito I. Role of nucleotide sequences of loxp spacer region in cre-mediated recombination. *Gene*. 1998;216:55-65
6. Endres BT, Staruschenko A, Schulte M, Geurts AM, Palygin O. Two-photon imaging of intracellular ca2+ handling and nitric oxide production in endothelial and smooth muscle cells of an isolated rat aorta. *J Vis Exp*. 2015:e52734

SUPPLEMENTAL MATERIAL

ONLINE FIGURE LEGENDS

Online Figure I. CD146 and NG2 expression dynamics in vascular smooth muscle development and the role of CD146 in cell differentiation. A, Descending aorta stained for CD31 (endothelium), NG2 and CD146 at E8.5, E10.5 and E16.5. Boxed areas are magnified. CD146 is expressed at E8.5 in the dorsal aorta endothelial cells. NG2 and CD146 expressing VSMCs appear at E10.5. Aortic smooth muscle cells maintain NG2 expression at E16.5 but downregulate CD146. CD146 expression is maintained in the endothelium (E), in microvasculature and smaller branches of the arteries (arrows). **B,** 10T1/2 cells upregulate SMMHC expression after 48 h exposure to TGFβ1. Stronger staining is evident in CD146 knockout cell lines C149 and C164. **C,** Staining for KI67 reveals more limited cell proliferation in C149 and C164 cell lines in comparison to wild type 10T1/2 cells. Scale bars: 50 μm.

Online Figure II. Characterization of CD146/*Mcam* genomic region and CD146-T2F:NG2-CRE-ERTM transgenic model. A, Epigenetic modifications from ENCODE database for mouse CD146/*Mcam* genomic region. Profiles are shown for 8 week old mouse cerebellum, heart, kidney, small intestine, lung and embryonic E14.5 limb. The DNA region, synthesized for CD146-T2F transgene, is shown underneath in blue. Histone modifications are indicated on the right together with CTCF binding motif. **B,** TAM administration at E14.5 leads by E17.5 to extensive labelling of CD146^{+}NG2^{+} mural cells by tdTomato (red) in double-transgenic CD146-T2F:NG2-CRE-ERTM mouse lung. Staining for native CD146 is shown in green, for NG2 in yellow and nuclei (Hoechst) in blue. **C,** At E12.5 tdTomato^{+} VSMCs in the aorta express PDGFRB (TAM injection at E10.5). **D,** Lymphatic vasculature (LYVE1, Lymphatic vessel endothelial hyaluronan receptor 1) is not labelled in the transgenic model (TAM injection at E14.5, analysis at E16.5). **E,** Determination of potential Lox-STOP-Lox cassette leakiness. CRE independent read-through of the STOP cassette can lead to endothelial labelling in the triple-transgenic strain since CD146 is highly expressed in endothelial cells. In the skeletal muscle we detected less than 4% of GFP labelled endothelial cells, whereas in the pancreas and lung only scarce GFP^{+} endothelial cells were evident (n=3; error bars: SD). Juv: TAM injection P6-P8, analysis at P22. Ad: 5 TAM injection after P35, analysis at P54. **D,** FACS plots illustrating CD31^{+}GFP^{+} cells in skeletal muscle (*Gastrocnemius*), pancreas and lung at P22. There is only limited leakiness of the STOP cassette and endothelial expression of GFP. Scale bars: B, 200 μm; C-D, 25 μm.

Online Figure III. Triple-transgenic model demonstrates dynamic reduction of CD146 expression in arterial smooth muscle maturation. A-D, VSMCs are visualized by SMA staining. **A,** Transgene expression was triggered at E14.5 and analyzed at E17.5. Only scattered tdTomato^{+}GFP^{+}SMA^{+} cells are evident in the thoracic aorta. **B,** At the same time tdTomato^{+}GFP^{+}SMA^{+} cells are more frequent in the abdominal aorta (quantified in Figure 3C). **C,** TAM administration in juvenile mice (P6-P8) labels only rare cells with GFP in the abdominal aortic wall by P22. **D,** TAM administration from P6 to P8 leads to efficient marking of arteriolar and microvascular mural cells in the kidney at P22. **E,** Very few cells are marked in the thoracic aorta when TAM was administered in adulthood (5 consecutive days) and analyzed 2 weeks after the last injection. TAGLN was used to identify aortic VSMCs. **F,** FACS plots illustrate decrease in tdTomato expression in adult hind limb skeletal muscles (see quantification in Figure 3I). **G,** NG2-CRE-ERTM:Rosa26-tdTomato mouse strain has very efficient VSMC labelling in the adult thoracic aorta. TAM was injected for 5 consecutive days starting at P35 and analyzed 2 weeks after the last injection. Single confocal plane is shown in all images. Scale bars: 50 μm.

Online Figure IV. Additional characterization of arterial branching site cells. A, TAM administration in juvenile growth period (P6-P8) leads to tdTomato and GFP labelling of progenitor cells at abdominal aorta inferior mesenteric artery branch site at P22. The cells form cushion-like structures close to CD31^{+} endothelium and vascular lumen. **B,** Second transgenic mouse line (BCD-146T2F) was

generated by independent pronuclear injection. This strain was crossed to NG2-CRE-ERTM line and thereafter to flippase reporter strain RCE-FRT. Postnatal TAM administration in the triple transgenic mice from P6 to P8 labelled progenitor cells at renal artery branch sites at P22 in tdTomato, whereas no red fluorescence was evident in the remaining aortic wall. The independent transgenic line confirms the existence of CD146 expressing progenitor cells at aortic branch sites. Single confocal plane is shown in all images. Scale bars: 50 µm.

Online Figure V. Characterization of VSMC turnover and the role of CD146 in VSMC adhesion. A, Descending aorta from E10.5, E16.5, P10 and 3 month old adult mice was stained for KI67 and SMA to identify proliferative VSMCs. Femoral artery from adult mouse is shown below. Only rare KI67 marked VSMCs can be seen in adult mouse thoracic aorta and femoral artery. **B,** Staining for activated caspase 3 (CASP3) did not reveal apoptotic VSMCs in the thoracic aorta. Note CASP3 positive cells in the surrounding tissue (boxed area is magnified). **C,** Cell adhesion assay was used to compare WT cells and CD146 knockout cells (C149, C164). Visualization of green fluorescent cells adhered to Matrigel before and after three PBS washes (details in Figure 5F-G). Scale bars: A-B, 50 µm; C, 200 µm.

Online Figure VI. Characterization of superficial femoral artery super-microanastomosis model. A-B, Artery was exposed, separated from the vein and nerve, clamped around the site of vessel division and repaired by super-microanastomosis (vessel repair <0.5mm Ø). **C,** Repair was performed in adult triple-transgenic mice, injected for 5 consecutive days with TAM before the surgery. Two weeks after super-microanastomosis pre-existing GFP^+ VSMCs have been replaced. GFP expression is still maintained in VSMCs (arrow) outside the regenerative zone. In the regenerating area GFP remains visible in pericytes of the microvasculature (arrowhead). Dotted line indicates the location of the artery. Confocal maximum intensity projection of the whole 3-dimensional stack is demonstrated. **D,** TAGLN (SM22)-CRE mouse was crossed to Rosa26-tdTomato reporter line enabling robust labelling of VSMCs (stained for SMA, arrow), but also fibroblasts surrounding the artery. **E,** 48 h after SFA super-microanastomosis the existing SMA and TAGLN-CRE:tdTomato marked VSMCs (arrow) have been lost. Scale bars: F, 1mm; C, 200 µm; D-E, 50 µm.

Online Figure VII. Adventitial cells respond to severe arterial injury and microanastomosis. In all images triple-transgenic mice were used. TAM was administered for 5 consecutive days and tissue was analyzed 2-3 weeks after the final injection. **A,** In control (unoperated) artery very limited CD44 expression is evident. Separate channels corresponding to Fig. 8F. **B,** In control artery SCA1 staining is limited to the adventitial layer (asterisk) and is absent from the *tunica media* (arrow). Separate channels corresponding to Fig. 8G. **C,** Similarly to SCA1, CD34 expression marks adventitial layer (asterisk) in control artery. **D,** 2 weeks following microanastomosis $CD34^+$ marked cells have penetrated the *tunica media* (arrow), replacing GFP^+ VSMCs. **E,** Three weeks after SFA wire induced injury $SCA1^+$ cells have not infiltrated *tunica media* (arrow). **F,** limited staining of CD44 is evident three weeks after SFA wire induced injury. Scale bars: 50 µm.

Online Figure I

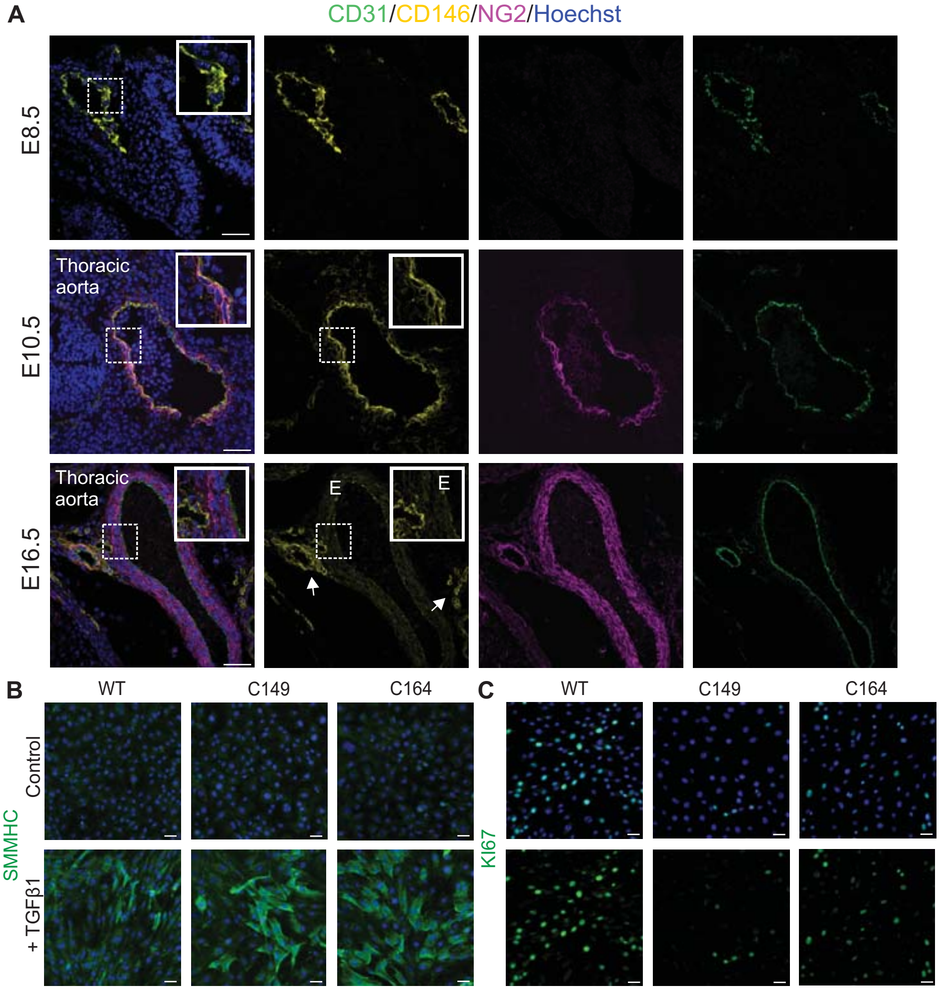

Online Figure II

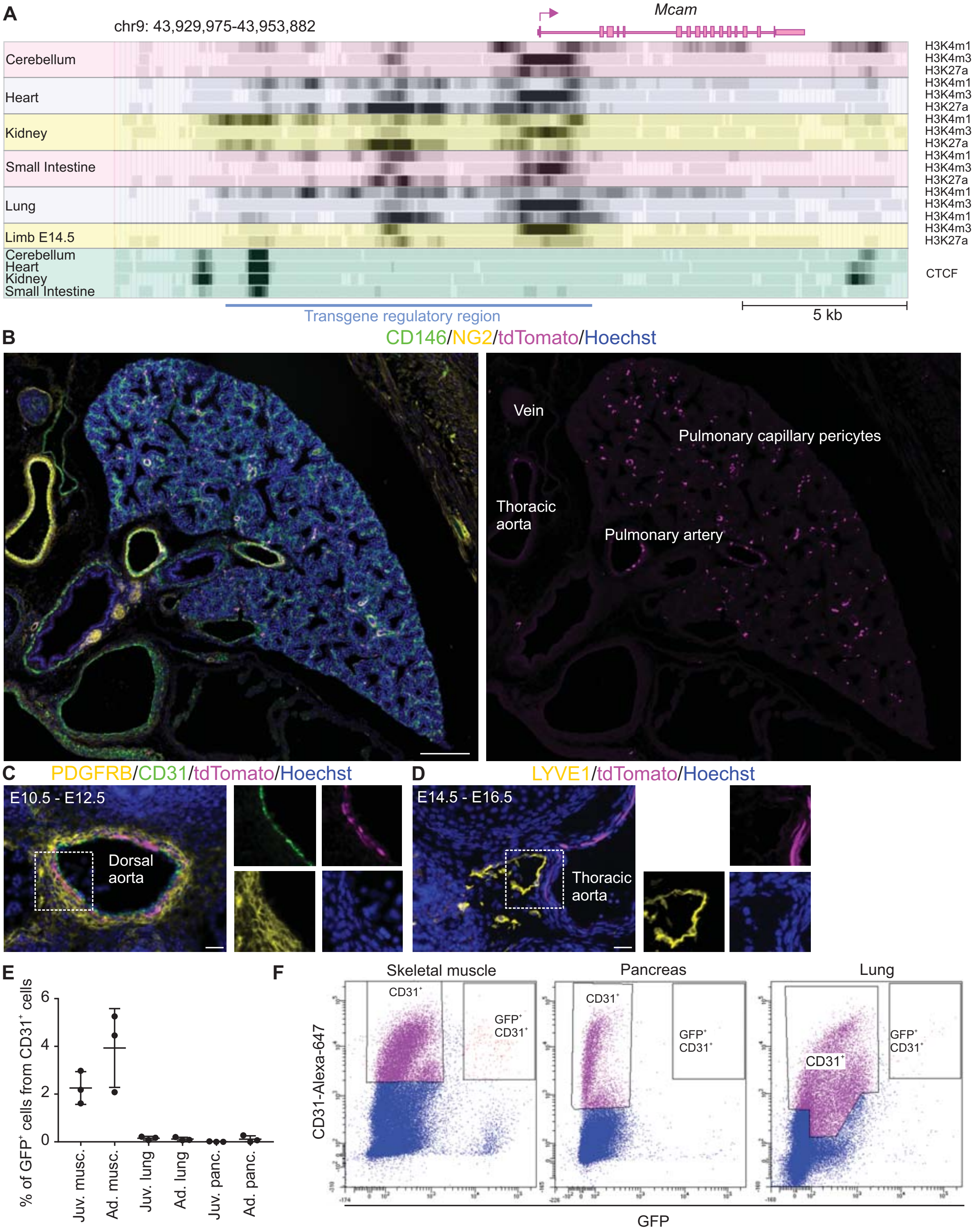

Online Figure III

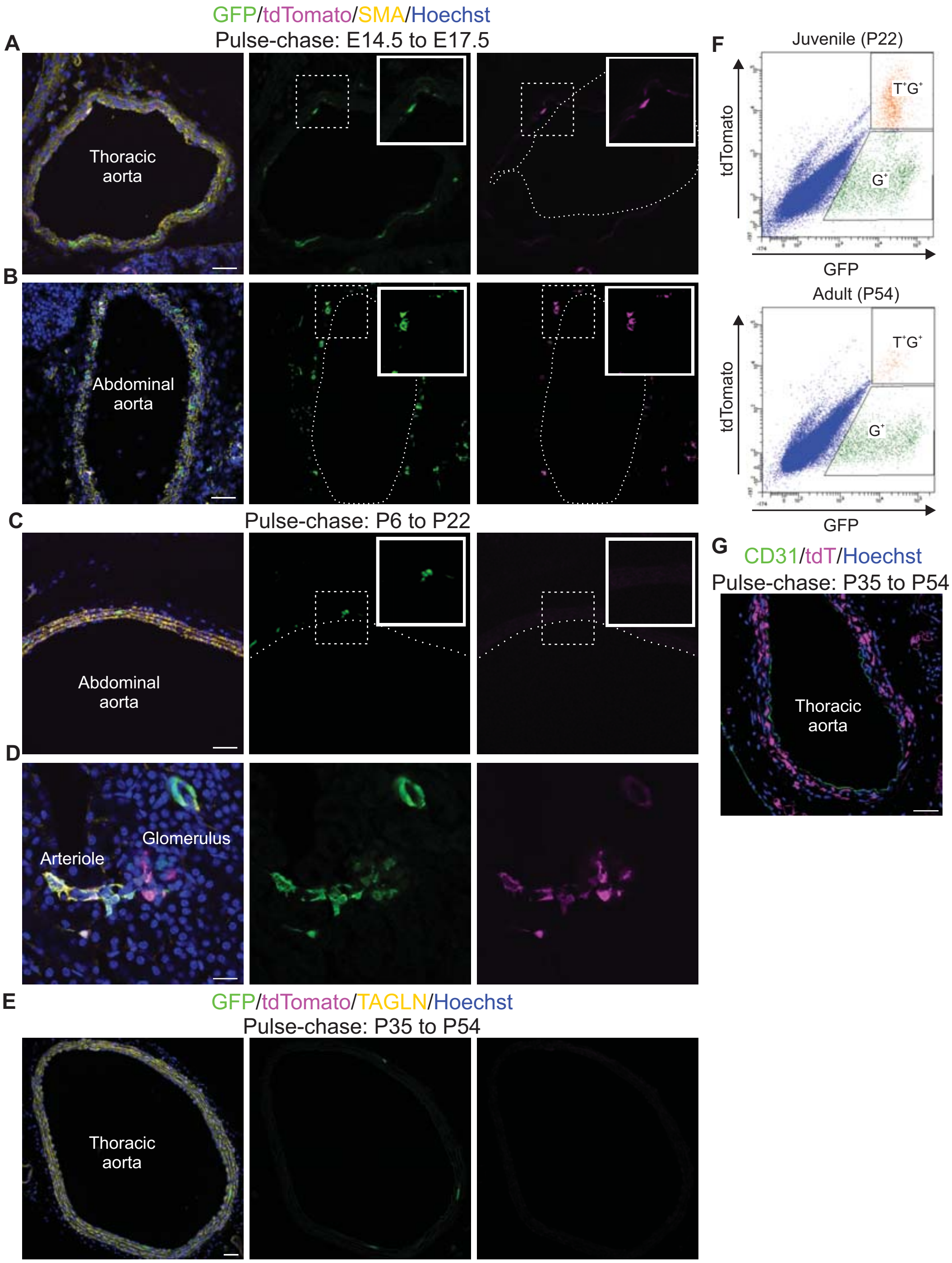

Online Figure IV

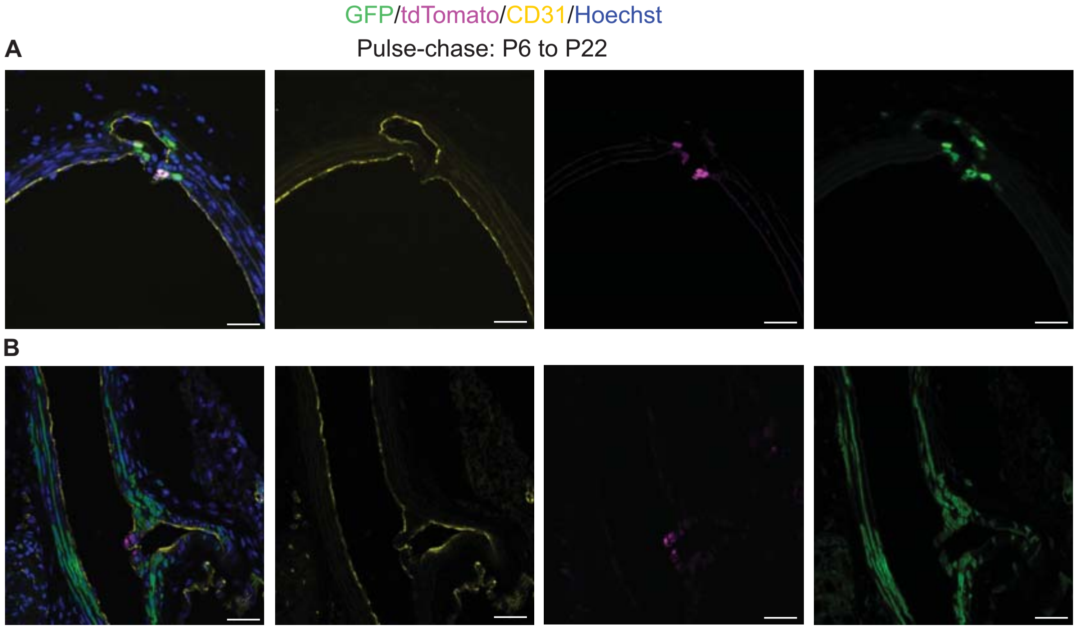

Online Figure V

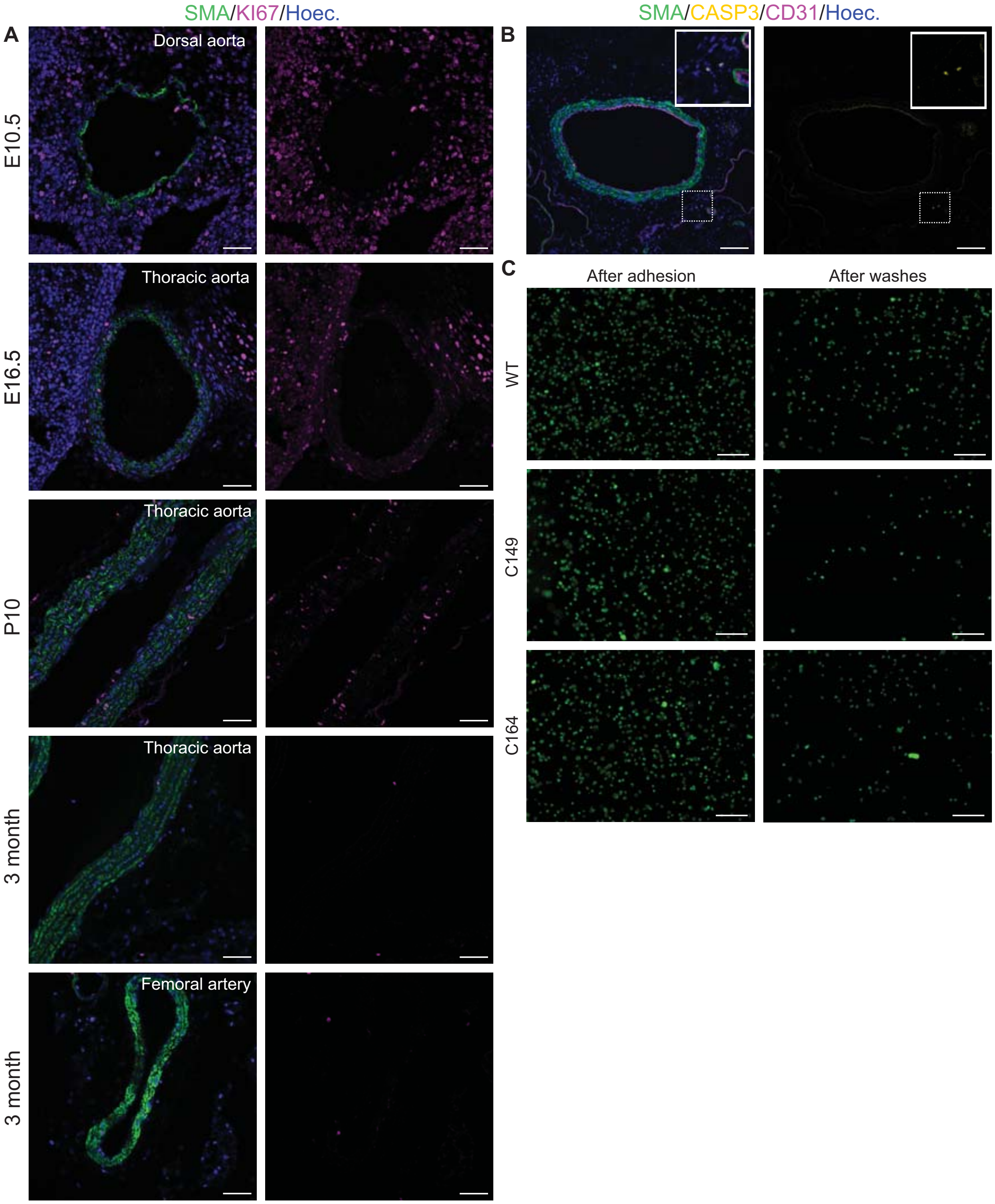

Online Figure VI

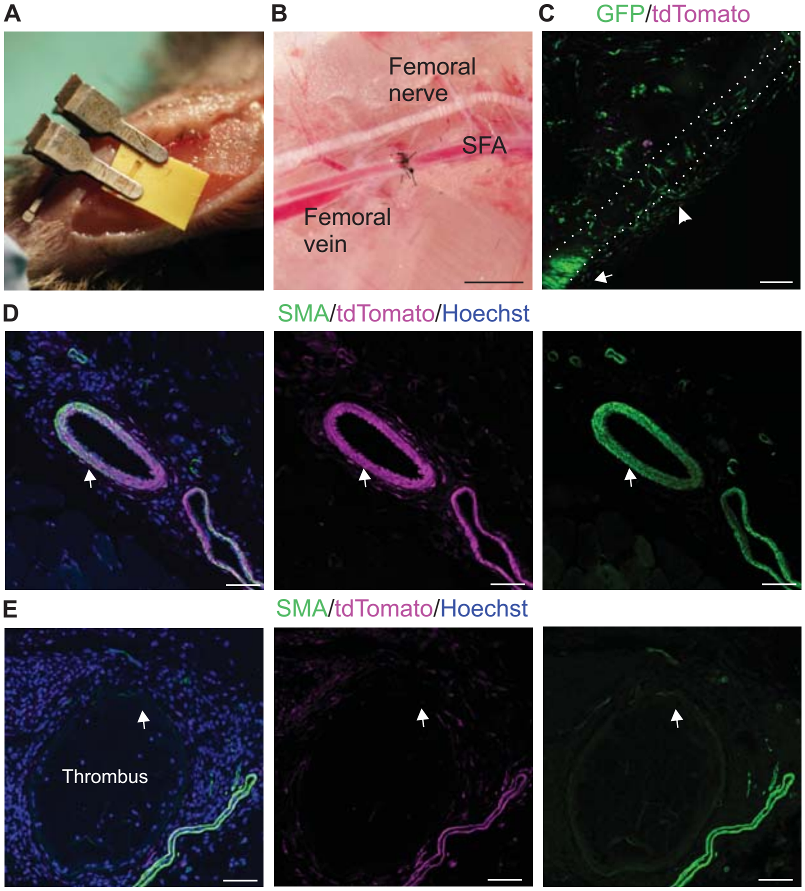

Online Figure VII

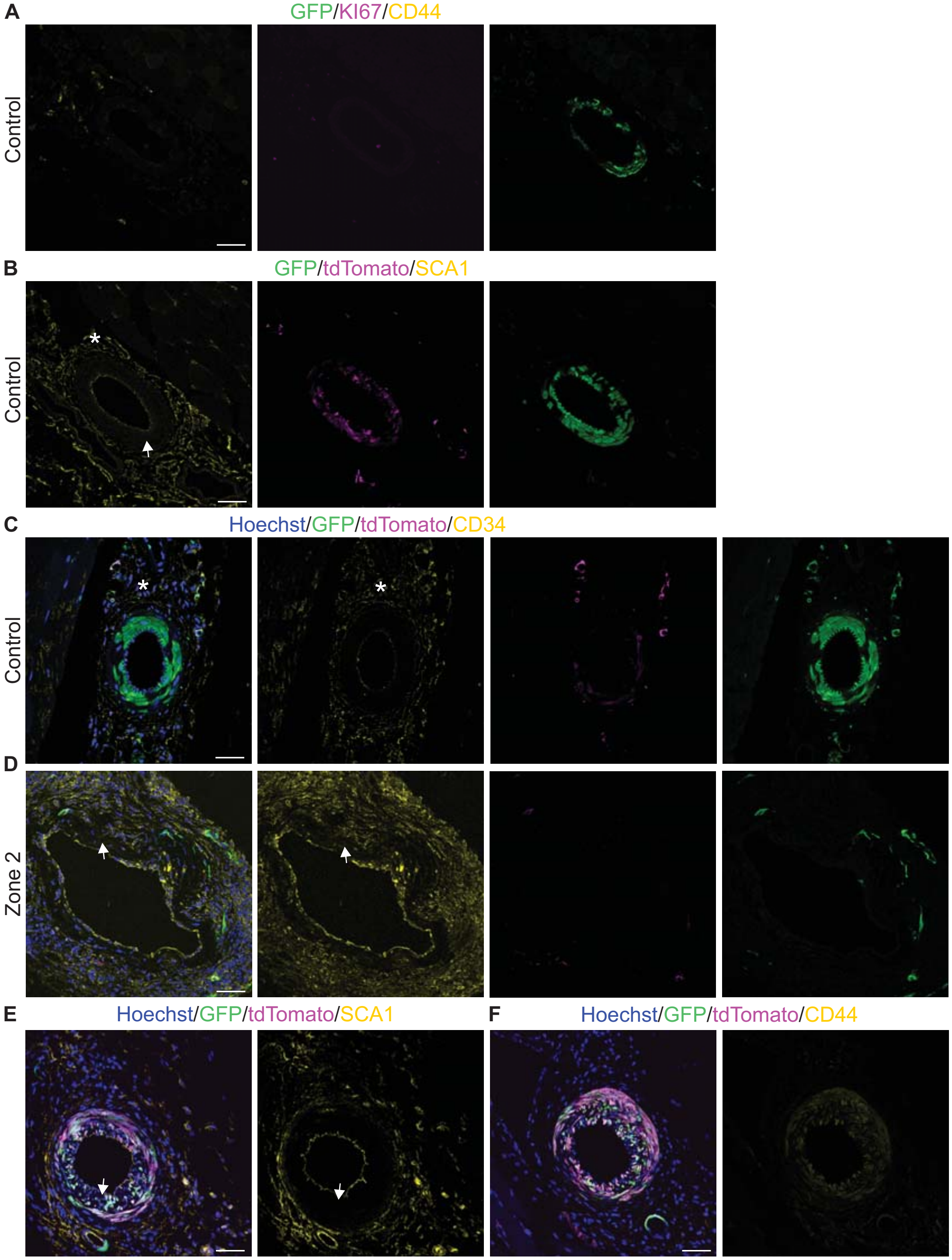

ONLINE TABLES

Online Table I. Upper and lower confidence intervals for relative normalized gene expression values (corresponding to Figure 1C).

	WT	WT+TGFβ1	C149	C149+TGFβ1	C164	C164+TGFβ1	Gene
**Lower 95% CI**	0.45	0.63	0.73	0.76	1.08	1.67	*Sma*
**Upper 95% CI**	0.68	0.90	1.29	2.17	1.81	1.95	
**Lower 95% CI**	0.35	1.07	0.55	1.21	1.29	2.68	*Tagln*
**Upper 95% CI**	0.46	1.39	1.60	2.50	1.78	3.67	
**Lower 95% CI**	0.90	1.26	0.84	0.47	1.25	0.59	*Pcna*
**Upper 95% CI**	1.13	1.60	1.87	0.89	1.79	1.52	

Online Table II. Statistical significance was analysed by Dunnett's test and the effect size as well as p-values are shown for comparison of untreated CD146 knockout cells (C149, C164) to untreated wildtype (WT) cells and for TGFβ1 treated CD146 knockout cell lines in comparison to corresponding TGFβ1 treated WT cells (corresponding to Figure 1C).

	Mean diff.	95% CI of diff.	p Value	Condition	Gene
**WT vs. C149**	-0.443	-0.7 to -0.187	0.0047	Untreated	*Sma*
**WT vs. C164**	-0.877	-1.133 to -0.62	0.0001		
**WT vs. C149**	-0.703	-1.101 to -0.306	0.0041	TGFβ1	
**WT vs. C164**	-1.043	-1.441 to -0.646	0.0005		
**WT vs. C149**	-0.67	-0.988 to -0.352	0.0017	Untreated	*Tagln*
**WT vs. C164**	-1.126	-1.443 to -0.808	0.0001		
**WT vs. C149**	-0.630	-1.081 to -0.179	0.0126	TGFβ1	
**WT vs. C164**	-1.947	-2.398 to -1.496	0.0001		
**WT vs. C149**	-0.34	-0.660 to -0.02	0.0399	Untreated	*Pcna*
**WT vs. C164**	-0.503	-0.824 to -0.183	0.0073		
**WT vs. C149**	0.753	0.460 to 1.046	0.0006	TGFβ1	
**WT vs. C164**	0.377	0.083 to 0.669	0.0183		

Online Table III. Loss of tdTomato labelling in aging was analysed by comparing juvenile (P22) skeletal muscle and lung to adult mouse (P54) corresponding tissues by FACS. Statistical significance was analysed by unpaired two-tailed t-test. Corresponding to Figure 3I.

	P22 muscle	P54 muscle	P22 lung	P54 muscle
**Mean**	0.832	0.120	0.042	0.014
**Std. Deviation**	0.135	0.08	0.008	0.012
**Lower 95% CI of mean**	0.497	-0.079	0.024	-0.015
**Upper 95% CI of mean**	1.167	0.319	0.061	0.042
**Difference between means**	-0.712 ± 0.091		-0.029 ± 0.008	
**95% CI of diff.**	-0.963 to -0.461		-0.051 to -0.007	
**p value**	0.0014		0.022	

Online Table IV. VSMC proliferation analysis. % of KI67^{+} VSMCs was quantified in the dorsal aortae of E10.5 mouse embryos and in the abdominal (AA) and thoracic (TA) aortae of E16.5, P10 and adult (AD, 3 months) mice, and in the branch sites of intercostal arteries from the aorta (BR) as well as in the superficial femoral arteries (SFA) of adult mice. Corresponding to Figure 5A.

	E10	E16 AA	E16 TA	P10 AA	P10 TA	AD AA	AD TA	AD BR	AD SFA
**Mean**	92.98	30.22	25.54	17.97	14.78	0.359	0.77	3.706	0.653
**Std. Deviation**	2.41	1.305	2.197	2.071	4.158	0.41	0.06	3.026	0.950
**Lower 95% CI of mean**	89.15	28.15	22.04	14.68	8.164	-0.29	0.675	-1.109	-0.225
**Upper 95% CI of mean**	96.82	32.3	29.03	21.27	21.4	1.01	0.865	8.522	1.532
**Dunnett's test**	E10 vs. E16 AA and TA								
**Difference between means (vs. E10)**		62.76 ± 1.512	67.45 ± 1.512						
**95% CI of diff.**		58.49 to 67.03	63.18 to 71.72						
**p value**		0.0001	0.0001						
**Two-tailed t-test**		E16 AA vs TA							
**Difference between means**		-4.687 ± 1.278							
**95% CI of diff.**		-7.814 to -1.561							
**p value**		0.0105							

Online Table V. Statistical analysis of cell adhesion of CRISPR-Cas9 edited cell lines. Cell adhesion was studied by fluorescence spectrometry and Dunnett's test was used to analyse significance between WT cells and mutant cells (C149 and C164). Corresponding to Figure 5G.

	WT	C149	C164
**Mean**	0.399	0.107	0.209
**Std. Deviation**	0.055	0.032	0.08
**Lower 95% CI of mean**	0.341	0.072	0.124
**Upper 95% CI of mean**	0.456	0.141	0.293
**Difference between means (vs. WT)**		0.292	0.19
**95% CI of diff. (WT, C149)**		0.209 to 0.375	
**95% CI of diff. (WT, C164)**			0.107 to 0.273
**p value (in comparison to WT)**		0.0001	0.000107

Online Table VI. Statistical analysis of GFP and tdTomato labelling in neointima cells 3 weeks after wire induced injury (mean % of total neointima cells; corresponding to Figure 6C).

	GFP^+tdT^+	GFP^+tdT^-
**Mean**	51.75	4.775
**Std. Deviation**	4.423	2.382
**Std. Error of Mean**	1.978	1.065
**Lower 95% CI of mean**	46.25	1.818
**Upper 95% CI of mean**	57.24	7.733

Online Table VII. SFA anastomosis causes VSMC proliferation. Statistical significance in comparison to unoperated SFA was analysed by unpaired two-tailed t-test (mean indicates % from total VSMCs; corresponding to Figure 8D).

	Unoperated control SFA	Anastomosis
**Mean**	0.653	22.56
**Std. Deviation**	0.950	3.987
**Lower 95% CI of mean**	-0.225	16.21
**Upper 95% CI of mean**	1.532	28.9
**Difference between means**	21.91 ± 1.523	
**95% CI of diff.**	18.46 to 25.35	
**p value**	0.0044	

Online Table VIII. SFA anastomosis causes adventitial cell proliferation. Corresponding to Figure 8E.

	% of KI67$^+$SCA1$^+$ cells	% of KI67$^+$CD44$^+$ cells
**Mean**	13.29	26.16
**Std. Deviation**	1.932	10.67
**Lower 95% CI of mean**	10.21	9.192
**Upper 95% CI of mean**	16.36	43.14
