# Supplementary material for: Distinct Cellular Mechanisms Underlie Smooth Muscle Turnover in Vascular Development and Repair
Source: Circ Res. 2017 Dec 12;122(2):267–81. doi: 10.1161/CIRCRESAHA.117.312111 (PMC5771686; doi:10.1161/CIRCRESAHA.117.312111)
Supplement: Supplementary file 2 [file res-122-267-s002.pdf]

Circulation Research

From: Emma Owen <Emma.L.Owen@manchester.ac.uk>
Sent: Monday, November 20, 2017 5:42 AM
To: CircRes@circresearch.org
Cc: Urmas Roostalu
Subject: Permission

Hi

This is to give permission for Urmas to acknowledge me by name.

Thanks

Emma

From: Peter Walker <peter.walker@manchester.ac.uk>
Sent: Monday, November 20, 2017 6:31 AM
To: CircRes@circresearch.org; Urmas Roostalu; Grace Bako
Subject: RE: paper accepted for publication - here with the attachment!

Dear Sir

This is to confirm that I give my permission to be acknowledge in Urmas's paper. The title is "Distinct cellular mechanisms underlie smooth muscle turnover in vascular development and repair".

Hi Urmas

Thank you for the acknowledgement.

Hi Grace

I presume you will have to send a similar email..

Pete Walker

Histology Facility Manager

Office: G.003b *(the office is swipe access controlled and behind the A V Hill Reception)*
Histology Core Facility | G.013 A V Hill building | Faculty of Biology, Medicine and Health | The University of Manchester | Oxford Road | Manchester | M13 9PT
Tel.+44(0)161 275 6771 Mobile ext. 07798 637041 Mobile (internal) 7751476

From: Urmas Roostalu
Sent: 20 November 2017 10:27
To: Peter Walker; Grace Bako
Subject: paper accepted for publication - here with the attachment!
Importance: High

Hi Pete and Grace,

Our manuscript has been accepted for publication at Circulation Research. Thank you for your advice and help! I would like to acknowledge you both by name. In order to do this could you please send an email to the editorial office, confirming that I have your permission to acknowledge you. It is their publication policy.

CircRes@circresearch.org

I would appreciate it if you could send it today as they want to proceed with the publication. I attach here the nearly final version of the paper. There will likely be press releases by the university and BBSRC in the coming weeks.

The title is "Distinct cellular mechanisms underlie smooth muscle turnover in vascular development and repair".

In case you fancy a drink we'll meet at Kro after work tomorrow.

Circulation Research

From: Peter March <P.March@manchester.ac.uk>
Sent: Monday, November 20, 2017 7:02 AM
To: CircRes@circresearch.org
Subject: Acknowledgement permission
Attachments: Roostalu2017-pre-accepted.pdf

Importance: High

Dear sir/madam,

I am delighted to give permission for my name to be used in the acknowledgments for the following paper:

“Distinct cellular mechanisms underlie smooth muscle turnover in vascular development and repair”.

Urmas Roostalu1, Bashar Aldeiri, Alessandra Albertini, Neil Humphreys, Maj Simonsen-Jackson, Jason KF Wong, Giulio Cossu

Thanks,

Peter

--
Dr Peter March
Bioimaging Senior Experimental Officer
Faculty of Biology, Medicine and Health
The University of Manchester
The Michael Smith Building
Oxford Road
Manchester
M13 9PT

Office: 0161 27 51571
Mobile (from a University phone): 77 51571 Mobile (from outside the University): 07747 118447

From: Michael Jackson <Michael.C.Jackson@manchester.ac.uk>
Sent: Monday, November 20, 2017 5:49 AM
To: CircRes@circresearch.org
Cc: Urmas Roostalu
Subject: "Distinct cellular mechanisms underlie smooth muscle turnover in vascular development and repair".

Hello,

I have been contacted by Dr Urmas Roostalu about being acknowledged in his paper -"Distinct cellular mechanisms underlie smooth muscle turnover in vascular development and repair".

I can confirm that I am happy to be acknowledged in the paper.

Thank you

Mike

Mike Jackson | Senior Technician - Flow Cytometry Core Facility | Faculty of Biology, Medicine and Health | University of Manchester | C3210 Michael Smith Building | Rumford Street | Manchester | M13 9PT
0161 275 5468 | internal 55468 |
Email: m.c.jackson@manchester.ac.uk
